# Neural correlates of emotional memory enhancement: The role of valence and arousal

**DOI:** 10.1162/IMAG.a.1213

**Published:** 2026-04-17

**Authors:** Ehssan Amini, David Coynel, Andreas Papassotiropoulos, Dominique J.-F. de Quervain

**Affiliations:** Division of Cognitive Neuroscience, Department of Biomedicine, University of Basel, Basel, Switzerland; Research Cluster Molecular and Cognitive Neurosciences, Department of Biomedicine, University of Basel, Basel, Switzerland; Psychiatric University Clinics, University of Basel, Basel, Switzerland; Division of Molecular Neuroscience, Department of Biomedicine, University of Basel, Basel, Switzerland

**Keywords:** episodic memory, emotional memory enhancement, emotional valence, emotional arousal, functional magnetic resonance imaging (fMRI), difference in memory (DM)

## Abstract

Emotional events are remembered better than neutral ones. While many human neuroimaging studies have identified brain regions involved, relatively few—and typically small—studies have disentangled how arousal and valence shape the neural substrates of this enhancement. We leveraged a large single-centre fMRI sample (n = 1,006) in which healthy young adults viewed negative, neutral, and positive pictures during scanning followed by an unannounced free-recall test. Using whole-brain subsequent-memory analyses (P_FWE_ < 0.05), we contrasted successful encoding of emotional (negative + positive) vs neutral items, then tested valence-specific effects (successful encoding: negative > neutral; positive > neutral), and finally controlled for subjective arousal via serial parametric modulation. Behaviourally, recall was higher for emotional than for neutral pictures. Consistent with prior meta-analytic evidence, emotional > neutral successful encoding engaged occipito-temporal visual cortex, anterior cingulate, insula, and amygdala. Additionally, we observed an extensive temporoparietal network, while hippocampal/parahippocampal activations were absent. After controlling for arousal, amygdala and insula effects were no longer significant, indicating these regions were sensitive to arousal rather than valence. Overlap of negative- and positive-valence enhancement localised primarily to the occipito-temporal cortex. Negative-specific enhancement recruited the lateral occipital/fusiform and bilateral supramarginal regions; positive-specific enhancement involved the rostral/caudal anterior cingulate, superior frontal, and parietal cortex, as well as the precuneus. Successful neutral encoding preferentially engaged frontoparietal control regions and bilateral lingual/parahippocampal cortex. Together, these findings dissociate valence-dependent from arousal-dependent mechanisms and reveal both partially overlapping and distinct networks for negative and positive memory enhancement, refining neurocognitive models of emotional memory encoding.

## 1 Introduction

Emotional experiences are typically remembered better than neutral ones, a phenomenon known as emotional memory enhancement (Kensinger, 2004; McGaugh, 2004). This enhancement plays a crucial role in survival and adaptation, as individuals are more likely to remember and avoid negative experiences while seeking out positive ones (McGaugh, 2013; Phelps, 2006). Decades of research have explored the mechanisms underlying the memory-enhancing effect of emotional arousal, beginning with lesion and pharmacological studies in animals, showing the crucial role of the amygdala (Gallagher et al., 1977; LaLumiere et al., 2003; McGaugh, 2004; Weiskrantz, 1956). Moreover, a study in patients with Urbach–Wiethe disease, who have bilateral amygdala damage, found no advantage in remembering emotional events over neutral ones in those patients, highlighting the amygdala’s central role in emotional memory enhancement (Cahill et al., 1995).

With the advent of functional magnetic resonance imaging (fMRI), it became possible to investigate the neural substrates of memory functions in healthy humans. A widely used paradigm to investigate successful memory encoding involves presenting participants with information during scanning, followed by a memory test for this information. This enables the identification of brain activity linked to subsequently remembered versus not remembered items, known as the difference in memory (DM) effect (Paller et al., 1987; Paller & Wagner, 2002). Dolcos et al. extended this framework, proposing that comparing DM for emotional versus neutral stimuli can reveal the neural substrates of emotional memory enhancement (Dolcos et al., 2004). This approach has been applied in numerous studies and has led to the identification of several brain regions associated with successful emotional memory encoding (Botzung et al., 2010; Dolcos et al., 2004; Kensinger & Schacter, 2008; Mickley & Kensinger, 2008; Thakral et al., 2022). Moreover, two meta-analyses reported brain regions consistently implicated in emotional memory enhancement, including the medial temporal lobe (amygdala, hippocampus, entorhinal cortex, perirhinal cortex, and parahippocampal cortex), bilateral visual processing areas (middle temporal gyrus, fusiform gyrus, and occipital cortex), bilateral temporal pole, orbitofrontal cortex, insula, putamen, and inferior and middle temporal gyri (Dahlgren et al., 2020; Murty et al., 2010).

However, while these findings provide valuable insights into the neural substrates of emotional memory enhancement in general, they mostly do not account for emotional components, in particular valence (its positive or negative nature) and arousal (the intensity of the emotional response). Understanding how these two dimensions influence memory encoding is crucial for unravelling the neural mechanisms underlying emotional memory enhancement (Kensinger, 2004).

To isolate the effects of arousal, Kensinger and Corkin (2004) presented participants with negative words varying in arousal level along with neutral words. Using a subsequent-memory analysis based on a recognition task, they found that high-arousal negative items were associated with increased amygdala activation, whereas low-arousal negative and neutral items engaged the inferior prefrontal cortex. Based on these findings, they proposed two partially distinct pathways for emotional memory enhancement: an arousal-driven amygdala-hippocampus network and a prefrontal-hippocampus network implicated in controlled, elaborative encoding (Kensinger & Corkin, 2004). Subsequent studies using similar encoding and recognition-based subsequent-memory paradigms have supported this model, demonstrating that encoding of high-arousal stimuli involves amygdala–hippocampal interactions and enhanced sensory processing, whereas encoding of low-arousal stimuli is more dependent on elaboration and semantic associations (Mickley Steinmetz & Kensinger, 2009; Sommer et al., 2008; Talmi et al., 2008).

While the above findings highlight the role of arousal in memory enhancement, the influence of emotional valence adds further complexity. Evidence suggests that the arousal’s impact on memory could be valence dependent, with amygdala connectivity to frontal and occipital regions being stronger during encoding of negative than during positive arousing stimuli (Mickley Steinmetz et al., 2010). Moreover, emotional memory enhancement for negative stimuli tends to rely more on temporo-occipital and sensory regions, while positive memory enhancement preferentially involves prefrontal areas (Botzung et al., 2010; Kark & Kensinger, 2015; Kensinger & Schacter, 2008; Mickley Steinmetz & Kensinger, 2009). Furthermore, Ritchey et al. demonstrated that during encoding, hippocampus-amygdala interactions were stronger for negative stimuli, while ventrolateral prefrontal cortex (vlPFC) –hippocampus connectivity was stronger for positive stimuli (Ritchey et al., 2011).

Despite these findings, a meta-analysis investigating valence-specific effects on successful memory encoding failed to identify consistent neural correlates (Dahlgren et al., 2020). This null result may be attributed to the limited number of studies included and the relatively small sample sizes used in many studies. Small sample sizes have often necessitated region-of-interest (ROI) analyses rather than whole-brain approaches or have led to suboptimal corrections for multiple comparisons in whole-brain analyses (typically using an uncorrected threshold of p < .001), which can hinder replication efforts and meta-analytic reliability (Button et al., 2013; Eklund et al., 2016; Turner et al., 2018).

To overcome these limitations, we leveraged a large-scale, single-centre fMRI dataset to investigate the neural correlates of emotional memory enhancement. The subjects viewed positive, negative, and neutral pictures inside the MRI scanner, followed by a free recall task outside the scanner. It is important to note that most previous studies have employed recognition-based memory tasks, whereas the present study used a free recall paradigm. We chose free recall because our primary aim was to test the enhancing effects of emotional arousal on episodic memory. Free recall places greater demands on self-initiated retrieval and the reinstatement of contextual information, providing a more stringent index of episodic memory. In contrast, recognition performance can be supported, at least in part, by non-episodic familiarity-based memory processes (Squire et al., 2007; Staresina & Davachi, 2006; Tulving, 2002). Hereby, first, we attempted to replicate the findings from the most recent meta-analysis (Dahlgren et al., 2020) using an emotional DM vs. neutral DM contrast. Next, we examined shared and distinct neural correlates of positive and negative emotional memory enhancement. Based on existing literature, we expected to observe activity in regions such as the amygdala for both emotional valences, valence-specific activation in frontal regions for positive stimuli, and greater temporo-occipital and sensory regions specific to negative stimuli. Moreover, given our large sample size, we anticipated identifying additional regions not previously reported. Furthermore, we investigated specific valence effects while controlling for arousal effects. We hypothesised that the activity of amygdala involved in emotional memory enhancement would be primarily driven by arousal and lose significance once arousal is controlled for. Finally, we also examined regions specifically associated with successful neutral memory encoding, expecting control and attention networks to be more engaged under conditions of low emotional salience.

## 2 Methods

### 2.1 Study sample

Data from 1,591 healthy young individuals (62.4% female; mean age ± SD = 22.4 ± 3.3 years) were gathered and used for behavioural analysis. However, to ensure sufficient statistical power for the first-level fMRI analysis, we included only participants who remembered at least five pictures per valence category (criterion 1). A total of 329 participants (20.7%) remembered fewer than 5 pictures per category and were, therefore, excluded. Furthermore, in fMRI analysis, to increase the signal-to-noise ratio when examining the neural correlates of emotional memory enhancement, we restricted the sample to participants who exhibited a behavioural emotional enhancement effect for both negative and positive valence categories (i.e., negative remembered—neutral remembered > 0 and positive remembered—neutral remembered > 0) (criterion 2). Of the initial 1,591 participants, 292 (18.36%) did not meet this criterion and were excluded (partially overlapping with participants not meeting criterion 1). Based on these inclusion criteria, data from 1,006 participants (63.1% female; mean age ± SD = 22.4 ± 3.1) were used for the group-level fMRI analyses (Supplementary Fig. S1). Because of the short time limit for the arousal rating task, some participants had missing values and were excluded from the parametric modulation analyses, resulting in a final sample of 792 participants (63.6% female; mean age ± SD = 22.4 ± 3.1 years) for these analyses. Participants were all healthy with no history of confirmed lifetime neuropsychiatric disorders and were not on any medications at the time of the study (except for hormonal contraceptives). All participants provided written informed consent, and the study protocol was approved by the Ethics Committee of Basel, Switzerland. These data have been used in several other studies, and parts of the Methods section are adapted from those studies (Geissmann et al., 2023; Petrovska et al., 2021; Spalek et al., 2015). Moreover, AI-assisted language models were utilised to improve the manuscript’s readability, coherence, and grammatical precision, while ensuring that the scientific content remained unchanged.

### 2.2 Task description

Seventy-two pictures, divided into 3 valence groups (negative, neutral, and positive), as well as 24 scrambled pictures, were presented. Pictures from the International Affective Picture System (IAPS) (Lang et al., 1997) were assigned to emotionally negative (mean ± SD = 2.3 ± 0.6), neutral (mean ± SD = 5.0 ± 0.3), and positive (mean ± SD = 7.6 ± 0.4) groups based on normative valence scores (scoring scale from 1: very negative to 5: neutral and 9: very positive). Negative and positive pictures were equated for valence extremity (p > .60). Mean normative arousal ratings were significantly higher for negative (mean ± SD = 5.9 ± 0.9) pictures than for both neutral (mean ± SD = 3.4 ± 0.5, p < .001) (neutral) and positive pictures (mean ± SD = 4.9 ± 0.8, p < .001) (scoring scale from 1: very low arousal to 9: very high arousal; Fig. 2). Eight neutral pictures were selected from an in‐house standardised picture set to match the picture set for content (human presence, animal/object, scenery). A chi-square test indicated no significant difference in the distribution of these content categories across valence groups (X^2^(4) = 4.73, p = .316). In addition, pictures did not differ in low level visual properties (mean luminance, RMS contrast, mean saturation, colourfulness, entropy, low-frequency energy, high-frequency energy, edge density) across valence categories (p-values ≥.1), except for SD luminance between neutral and negative categories: p < .05, Supplementary Figure S4. Entropy, edge density, and high-frequency energy served as proxies for visual complexity.

**Fig. 1.**
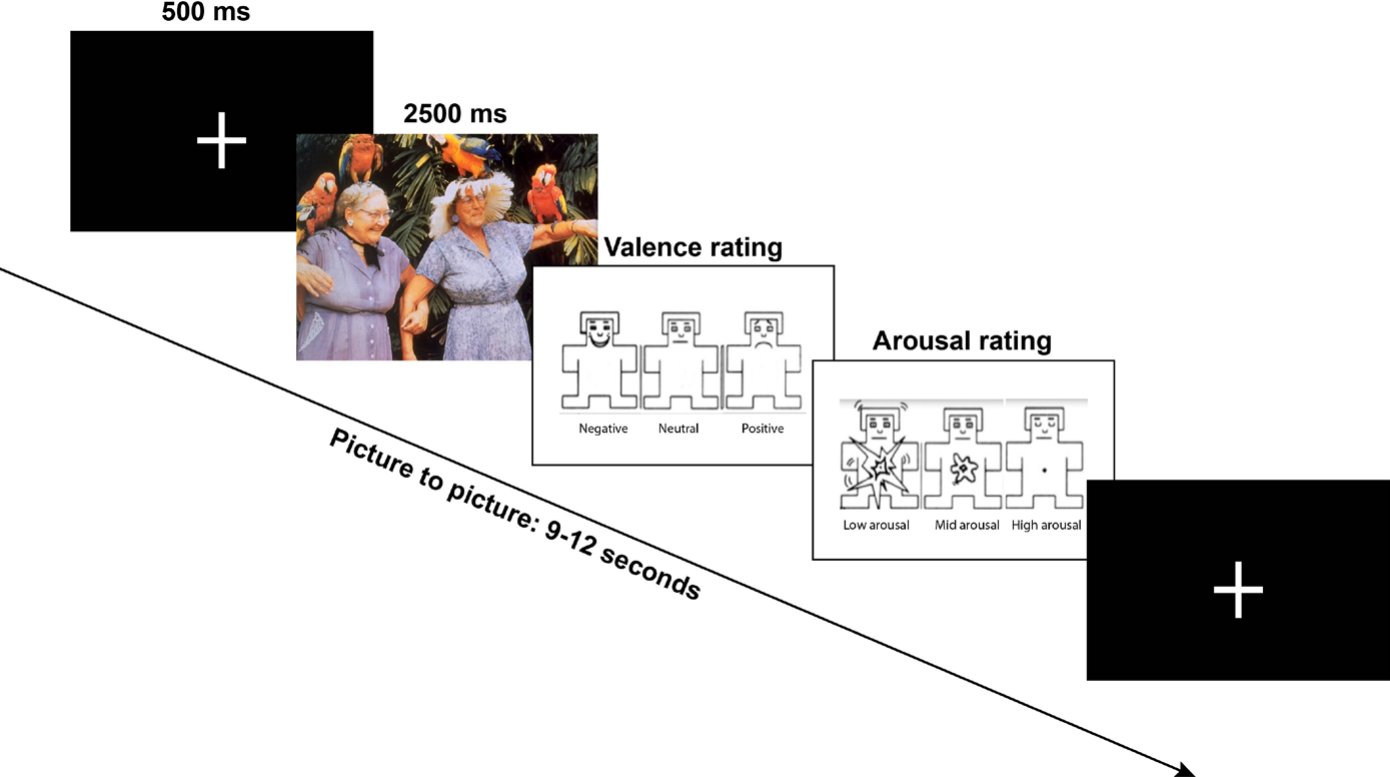
Schematic of the picture-encoding task. Each trial began with a fixation cross (500 ms), followed by the stimulus presentation (2,500 ms). Participants then rated each image for emotional valence and arousal using a 3-point visual scale.

Examples of pictures included erotica, sports, and appealing animals (positive); bodily injury, snake, and attack scenes (negative); and neutral faces, household objects, and buildings (neutral). Pictures were presented in an event‐related design for 2.5 seconds in a quasi‐randomised order with a maximum of four pictures of the same category shown consecutively. A fixation cross appeared on the screen for 500 ms before each picture presentation. Trials were separated by a variable inter‐trial period (the period between the appearance of a picture and the next fixation cross) of 9–12 seconds (jitter). During the inter-trial interval, participants rated each picture on two dimensions using two separate three-point scale via button press: valence (negative, neutral, positive) and arousal (low, medium, high) (Fig. 1). Prior to the task, participants were instructed as follows: “Please look carefully at the images and let them sink in. Try to place yourself into the depicted scene and empathize with it. After each image, you will be asked to rate the image on a 3-point scale along two different dimensions: Emotionality and Arousal.” For each participant the average arousal and valence rating per valence category were calculated and considered as subjective valence/arousal rating for each category. Four additional neutral pictures were added to the main set of 72 stimuli to control for primacy and recency effects in memory. These “primacy and recency” pictures were not included in the main 72-picture set and served solely to minimise position-related biases in recall performance. The scrambled pictures consisted of a geometrical object in the foreground with the background containing the colour information of all pictures used in the experiment (except primacy and recency pictures), overlaid with a crystal and distortion filter (Adobe Photoshop CS3). The object had to be rated regarding its form (vertical, symmetric, horizontal) and size (small, medium, large).

In an unannounced recall task outside of the scanner, subjects were asked to freely recall the previously presented pictures 10 minutes after the end of picture encoding. An unannounced free recall test was used to avoid recall performance being influenced by individual differences in learning strategies. Participants wrote down a short description (a few words) of the recalled pictures. There was no time limit for this task. No details were required for correct scoring, as the pictures were all distinct from each other. Two trained investigators independently rated the descriptions for recall success (inter-rater reliability > 98%). If a picture was rated differently by two raters, a third blinded rater made a final decision. The total and per-valence number of freely recalled pictures (excluding primacy, recency, and training ones) was defined as the free recall performance.

Behavioural variables were analysed separately for memory performance, subjective arousal ratings, and subjective valence ratings. For each outcome, paired-samples t-tests were used to compare performance across valence categories (neutral, negative, positive). The results were controlled for familywise error rate using the Bonferroni correction method.

To assess potential effects of emotional valence on retrieval accessibility, recall output order was analysed. For each participant, the serial position at which each image was recalled during the free recall task was recorded. For each image, a mean recall position was then calculated by averaging its recall position across all participants who recalled that image. These mean recall positions were subsequently compared across valence categories to determine whether positive, negative, or neutral images differed in their average retrieval order.

To examine whether memory performance varied as a function of image content across valence categories, we conducted a trial-level item analysis. Specifically, a logistic mixed-effects model predicting subsequent memory performance (remembered vs. not remembered) from valence category, content category (human, animal/object, scenery), and their interaction was fitted. Random intercepts for subjects and items were included to account for individual differences in memory performance and baseline differences in item memorability.

To account for potential effects of mood and anxiety on emotional memory performance, participants completed the self-administered version of Montgomery–Åsberg Depression Rating Scale (MADRS-S) to assess depressive symptoms (Svanborg & Asberg, 2001), as well as the State–Trait Anxiety Inventory (STAI), which measures both state and trait anxiety (Laux et al., 1981), on the day of testing. Associations between mood scores (MADRS-S, STAI-state, and STAI-trait) and free recall performance were assessed using linear mixed-effects models with stimulus valence as a fixed effect and participant as a random intercept.

### 2.3 fMRI data gathering and preprocessing

#### 2.3.1 Data acquisition

A high-resolution T1-weighted anatomical image was acquired using a magnetization prepared gradient echo sequence (MP-RAGE, TR = 2000 ms; TE = 3.37 ms; TI = 1000 ms; flip angle = 8; 176 slices; FOV = 256 mm; voxel size = 1 × 1 × 1 mm^3^). All functional images were acquired on the same Siemens Magnetom Verio 3 T whole-body MR scanner equipped with a 12-channel head coil. Blood oxygen level-dependent fMRI was acquired using a single-shot echoplanar sequence along with generalised auto-calibrating partially parallel acquisition (GRAPPA), using the following parameters: echo time (TE) = 25 ms, field of view (FOV) = 22 cm, acquisition matrix = 80 × 80 (interpolated to 128 × 128, voxel size = 2.75 × 2.75 × 4 mm^3^), acceleration factor = 2, flip angle alpha = 82°. We used an ascending interleaved sequence with a repetition time (TR) = 3,000 ms, measuring 32 contiguous axial slices that were placed along the anterior-posterior commissure plane based on a mid-sagittal scout image. To minimise scanner noise, all subjects wore earplugs and headphones. They were instructed to remain motionless, with small foam pads used for additional head fixation. MR-compatible LCD goggles (VisualSystem, NordicNeuroLab) were employed to present behavioural tasks, with vision correction applied when necessary.

#### 2.3.2 Preprocessing

fMRI data were pre-processed using SPM12 (Statistical Parametric Mapping, Wellcome Trust Centre for Neuroimaging; http://www.fil.ion.ucl.ac.uk/spm/) within MATLAB R2016b (MathWorks). Volumes were slice-time corrected to the first slice (acquired at TR/2), realigned using the “register to mean” option, and co-registered to the anatomical image by applying a normalised mutual information 3-D rigid-body transformation. Successful co-registration was visually verified for each subject. Using Dartel and templates from structural data (see structural MRI preprocessing), subject-to-template and template-to-MNI transformations were combined to map the functional images to MNI space. The functional images were smoothed with an isotropic 8 mm full-width at half-maximum (FWHM) Gaussian filter. Normalised functional images were masked using information from their respective T1 anatomical image as follows. At first, the three-tissue classification probability maps of the “Segment” procedure (grey matter, white matter, and cerebrospinal fluid) were summed to define the brain mask. This mask was binarised, dilated, and eroded with a 3 × 3 × 3 voxel kernel using fslmaths from FSL (Jenkinson et al., 2012) to fill in potential small holes. The previously computed DARTEL (Ashburner, 2007) flow field was used to normalise the brain mask to MNI space at the spatial resolution of the functional images. The resulting non-binary mask was thresholded at 50% and applied to the normalised functional images. Consequently, the implicit intensity-based masking threshold usually employed to compute a brain mask from the functional data during the first level specification (spm_get_defaults(“mask.thresh”), by default fixed at 0.8) was not needed any longer and set to a lower value of 0.05.

### 2.4 fMRI analysis

All fMRI analyses were conducted using SPM12 within MATLAB R2021b (MathWorks). Visualisation of results was performed using MRIcroGL (Rorden, 2025a), and Surf Ice (Rorden, 2025b).

**Fig. 2.**
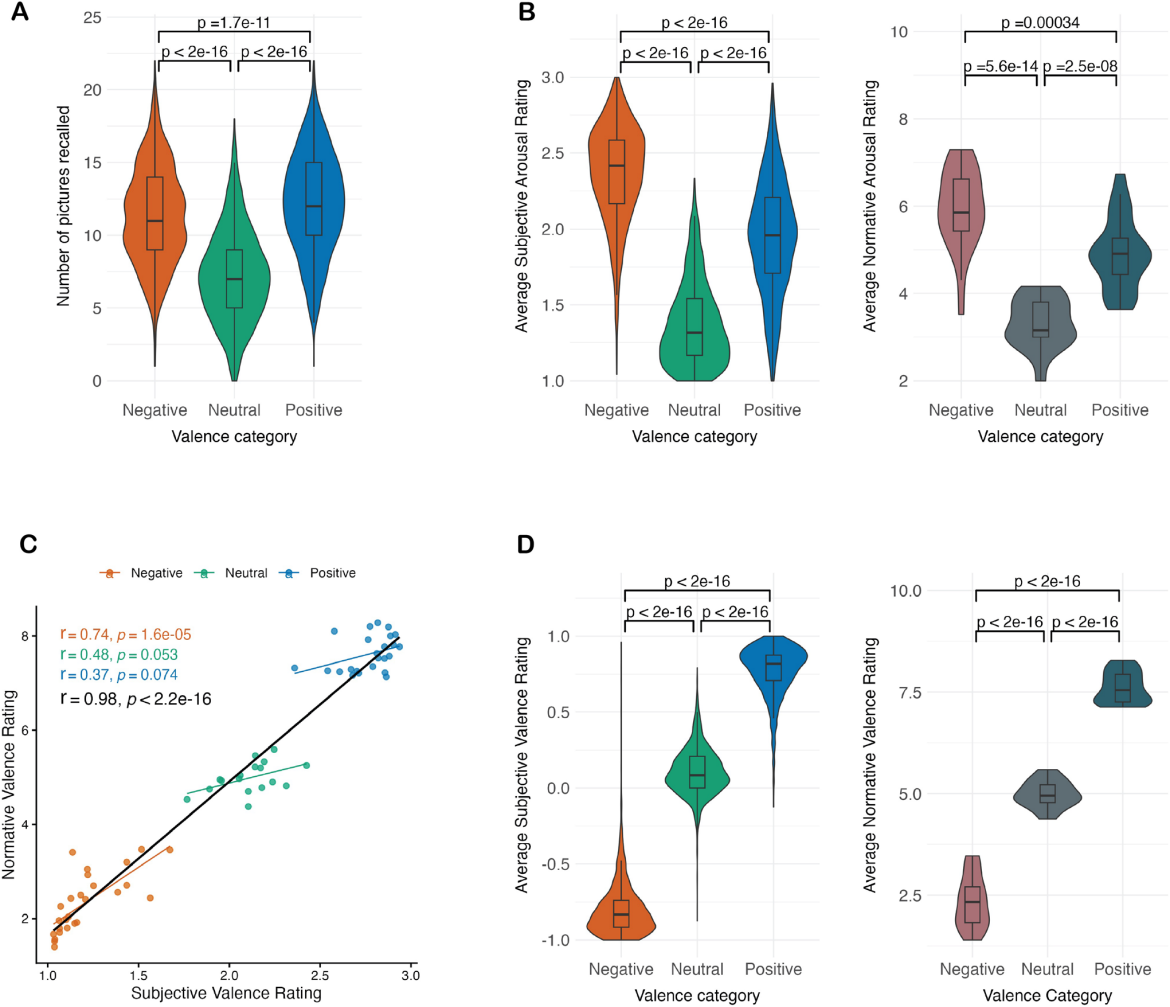
Distribution of behavioural variables. (A) Number of remembered pictures per valence category. (B) Subjective arousal ratings per valence category in the left (1 = low arousal, 3 = high arousal) and IAPS normative arousal rating per valence category in the right (1 = low arousal, 9 = high arousal). (C) Correlation between IAPS normative valence rating and subjective valence rating in the current study. The x-axis represents the average valence rating across all subjects (1 = negative, 2 = neutral, and 3 = positive) and the y-axis represents the normative valence rating for each picture (1 = negative, 5 = neutral, and 9 = positive). Correlations are shown per valence category and in total. (D) Subjective valence ratings per valence category in the left (-1 = negative, 0 = neutral and 1 = positive) and IAPS normative valence rating in the right (1 = negative, 5 = neutral, and 9 = positive). P-values in panels A, B, and D are based on Bonferroni-corrected paired t-tests. The correlation in panel C is based on Pearson’s r.

It should be noted that in all fMRI analyses, emotional valence categories (negative, neutral, positive) were defined based on normative valence ratings from the International Affective Picture System (IAPS) to ensure consistency of stimulus classification across participants and to preserve matching of low-level visual features and content across valence categories. Although individual differences in emotional interpretation can be expected, normative valence categorisation showed a strong correspondence with participants’ subjective valence ratings at the population level (Fig. 2C).

In contrast, subjective arousal ratings were used in the analyses, as arousal is known to vary substantially across individuals. Subjective arousal was, therefore, incorporated using parametric modulation, allowing us to account for arousal-related variability in neural responses independently of valence.

#### 2.4.1 First-level fMRI analysis

Intrinsic autocorrelations were accounted for using a first-order autoregressive model AR (1), and low-frequency drifts were removed via a high-pass filter (time constant: 128 seconds). For each subject, evoked haemodynamic responses to event types with zero duration (e.g., button presses) were modelled using a delta function, whereas events with a nonzero duration (e.g., picture presentation) were modelled using a boxcar function (duration: 2.5 seconds). Each event was convolved with a canonical haemodynamic response function (HRF).

#### 2.4.2 Emotional memory enhancement

In the first-level analysis of emotional memory enhancement, positive and negative pictures were combined into a single emotional category, and four main regressors were defined: emotional remembered, emotional not remembered, neutral remembered, and neutral not remembered. The scrambled picture category, button presses, and rating scale presentation during the ratings were modelled separately. Six movement parameters from spatial realignment were included as regressors of no interest. The subject-specific contrasts of interest were the contrast between emotional DM (emotional remembered > emotional not remembered) and neutral DM (neutral remembered > neutral not remembered), as well as their reverse contrast (neutral DM > emotional DM), used to identify regions more activated during the successful encoding of neutral events compared with emotional ones.

The group-level analyses investigated the average activation of the contrasts of interests, considering the following covariates: sex, age, and batch effects (two MR gradient changes, one MR software upgrade, and the room in which subjects completed the free recall task). Whole-brain analyses were performed using a two-tailed FWE-corrected threshold of P_FWE_ < 0.05. A minimum cluster size of five voxels was applied for visualisation and reporting purposes only and was not used for statistical inference.

#### 2.4.3 Valence-specific emotional memory enhancement

For valence-specific subsequent memory effect models at the first level, pictures were categorised into six valence * memory conditions (positive remembered, positive not remembered, neutral remembered, neutral not remembered, negative remembered, and negative not remembered). Rating scales presentations, scrambled pictures presentation, and button presses were modelled separately, and six movement parameters from spatial realignment were included as regressors of no interest.

To estimate valence-specific emotional memory enhancement, a two-step contrast approach was used. First, the DM was estimated for each valence category by contrasting remembered vs. not-remembered pictures, resulting in negative DM, positive DM, and neutral DM contrasts. In the second step, the negative DM and positive DM contrasts were each compared against the neutral DM contrast. The resulting t-maps were used in the group-level analyses, controlling for age, sex, and batch effects.

Whole-brain analyses were performed using a two-tailed FWE-corrected threshold of P_FWE_ < 0.05 and a minimum cluster size of five voxels. To identify shared brain regions involved in both negative and positive emotional memory enhancement, the two contrasts were overlaid using the inclusive mask function in SPM. Similarly, distinct regions were identified using the exclusive mask function. Finally, the reverse contrast (neutral DM > negative/positive DM) was used to identify brain regions more activated in neutral successful memory encoding compared with either negative or positive encoding.

#### 2.4.4 Valence-specific emotional memory enhancement controlled for arousal

To control for arousal effects, a parametric modulation (PM) analysis was performed. The general linear model for each subject included regressors for picture presentation of the three main valence categories (positive, neutral, and negative) and two parametric modulators per picture category with the following order: (1) subjective arousal rating for each picture (arousal-PM; 1 = low, 2 = medium, 3 = high) and (2) whether the picture was later remembered (memory-PM; 1 = remembered, 0 = not remembered). Parametric modulators were entered with serial orthogonalisation, such that the memory-PM captured variance not explained by the arousal-PM. The scrambled picture category, button presses, and rating scale presentation during the ratings were modelled separately, and six movement parameters from spatial realignment were included as regressors of no interest.

In this context, the memory-PM regressor captures memory-related variability of the BOLD response that is (1) not explained by the canonical HRF (mean activation) and (2) not explained by variability due to subjective arousal ratings.

To estimate valence-specific emotional memory enhancement activation controlled for arousal, a contrast was computed between memory-PM for either the negative or positive category against neutral memory-PM. Whole-brain analyses were performed using a two-tailed FWE-corrected threshold of P_FWE_ < 0.05 and a minimum cluster size of five voxels. To identify shared brain regions involved in both negative and positive emotional memory enhancement, the two contrasts were overlaid using the inclusive mask function in SPM. Distinct regions were identified using the exclusive mask function in the same manner. Finally, the reverse contrast (neutral memory-PM > negative/positive memory-PM) was used to identify brain regions more activated in neutral successful memory encoding compared with either negative or positive encoding, controlling for subjective arousal ratings.

#### 2.4.5 ROI analyses

To better characterise the average activity across conditions, we conducted region-of-interest (ROI) analyses on the clusters identified in the whole-brain contrast analyses. These analyses allowed us to quantify the direction and magnitude of effects at the regional level. For each region of interest (ROI) identified in the analyses above, the average beta coefficients for the regressors or contrasts of interest were extracted across all voxels within the cluster using the *get_marsy* function within the Marsbar 0.45 toolbox (Brett et al., 2002). These values were then visualised across the voxels and population using the ggplot2 package (Wickham & Sievert, 2009) in Rstudio version 4.3.2 (Allaire, 2012; Team, 2016). This analysis provides a clearer picture of the average brain activity within each ROI, helping to localise the source of the signal.

#### 2.4.6 Construction of a population-average anatomical probabilistic atlas and cluster labelling

Automatic segmentation of the subjects’ T1-weighted images was used to build a population-average probabilistic anatomical atlas. More precisely, each participant’s T1-weighted image was first automatically segmented into cortical and subcortical structures using FreeSurfer (version 4.5, http://surfer.nmr.mgh.harvard.edu/) (Fischl et al., 2002). Labelling of the cortical gyri was based on the Desikan–Killiany Atlas (Desikan et al., 2006), yielding 35 regions per hemisphere. We also labelled 28 subcortical regions in total (11 subcortical bilateral regions and 6 central regions comprising corpus callosum and brainstem) following Fischl et al. (2002). The segmented T1 image was then normalised to the study-specific anatomical template space using the subject’s computed warp field and affine-registered to the MNI space. The normalised segmentations were finally averaged across subjects to create a population-averaged probabilistic atlas. Each voxel of the template could consequently be assigned a probability of belonging to a given anatomical structure based on the individual information from 1,000 subjects, which were part of the subjects included in the present study. This in-house atlas was later used in combination with the *atlas query* function within the FSL framework (Jenkinson et al., 2012) to label the clusters found in group-level analysis based on the location of the peak voxels in each cluster.

To compare ROIs with resting-state networks, we used the Schaefer atlas with 100 parcellations and 7 networks (Schaefer et al., 2018). Each ROI’s peak location was visually inspected for overlap with the atlas networks and labelled accordingly. For larger ROIs that spanned multiple networks, all relevant network labels were included.

#### 2.4.7 Comparison with meta-analytic findings

To contextualise our findings, we compared the results of our whole-brain contrast maps with those reported in the most recent coordinate-based meta-analysis on emotional memory encoding (Dahlgren et al., 2020). Seed-based d-mapping (SDM) results of the study were downloaded from the Neurovault repository (https://identifiers.org/neurovault.collection:6627) and laid over our results for comparison.

## 3 Results

### 3.1 Behavioural results

Behavioural analyses were conducted in the full sample (N = 1,591).

Participants recalled on average 30.8 ± 8.3 out of 72 pictures. Negative pictures (11.4 ± 3.3) were remembered more frequently than neutral pictures (7.2 ± 3.2; t_(1579)_ = 51.08, p < .001, 95% CI [3.95, 4.27], Cohen’s d [95% CI] = 1.26 [1.19, 1.32]), but less frequently than positive pictures (12.2 ± 3.4; t_(1579)_ = -10.31, p < .001, 95% CI [-0.99, -0.68], Cohen’s d [95% CI] = -0.25 [-0.29, -0.20]). Positive pictures were also remembered more frequently than neutral pictures (t_(1579)_ = 61.82, p < .001, 95% CI [4.79, 5.10], Cohen’s d [95% CI] = 1.49 [1.42, 1.56]; Fig. 2A).

Participants’ subjective valence ratings were strongly aligned with the IAPS normative ratings (correlation: r = 0.98, p < .001) confirming that participants’ valence ratings closely tracked the normative emotional categories of the images (Fig. 2C). Participants’ subjective valence ratings differed reliably across picture categories. Negative pictures (-0.8 ± 0.18) were rated as more negative than neutral pictures (0.1 ± 0.16; t_(1578)_ = -145.23, p < .001, 95% CI [-0.91, -0.89], Cohen’s d [95% CI] = -5.23 [-5.50, -4.96]), whereas positive pictures (0.77 ± 0.17) were rated as more positive than neutral pictures (t_(1578)_ = 132.19, p < .001, 95% CI [0.66, 0.68], Cohen’s d [95% CI] = 4.02 [3.84, 4.2]; Fig. 2D).

Importantly, participants’ subjective arousal ratings differed across picture categories in line with IAPS normative ratings. Negative pictures (2.36 ± 0.32) were rated as more arousing than neutral pictures (1.37 ± 0.27; t_(1578)_ = 126.13, p < .001, 95% CI [0.98, 1.01], Cohen’s d [95% CI] = 3.32 [3.18, 3.45]). Negative pictures were also rated more arousing than positive pictures (1.94 ± 0.37; t_(1578)_ = 49.64, p < .001, 95% CI [0.40, 0.44], Cohen’s d [95% CI] = 1.2 [1.13, 1.26]), indicating that arousal was not matched between negative and positive categories. Positive pictures were also rated as more arousing than neutral pictures (t_(1578)_ = 75.58, p < .001, 95% CI [0.56, 0.59], Cohen’s d [95% CI] = 1.69 [1.62, 1.76]; Fig. 2B).

Analysis of recall output order revealed that positive images were recalled earlier than neutral images, indicating greater retrieval accessibility; however, there was no significant difference in recall position between positive and negative images. Thus, differences in retrieval accessibility do not appear to explain the observed valence-related differences in encoding-related subsequent memory effects between positive and negative categories (Supplementary Fig. S3).

Mood/anxiety scores showed variability in depressive symptoms (mean MADRS-S ± SD = 7.4 ± 5.47), trait anxiety (mean STAI-T ± SD = 36.0 ± 7.9), and state anxiety (mean STAI-S ± SD = 33.5 ± 6.22). Higher levels of depressive symptoms (t_(3005)_ = −2.34, p = .019; β [95% CI] = −0.05 [-0.09, -0.01]), trait anxiety (t_(2996)_ = −2.51, p = .012; β [95% CI] = −0.05[-0.09, -0.01]), and state anxiety (t_(2993)_ = −2.84, p = .005; β [95% CI] = −0.06 [-0.10, -0.02]) were each associated with a small reduction in overall free recall performance. Importantly, none of the mood/anxiety measures showed a significant interaction with stimulus valence in predicting free recall (all F’s ≤ 2.20, all p’s ≥ .11).

### 3.2 Neural correlates of emotional memory enhancement

**Fig. 3.**
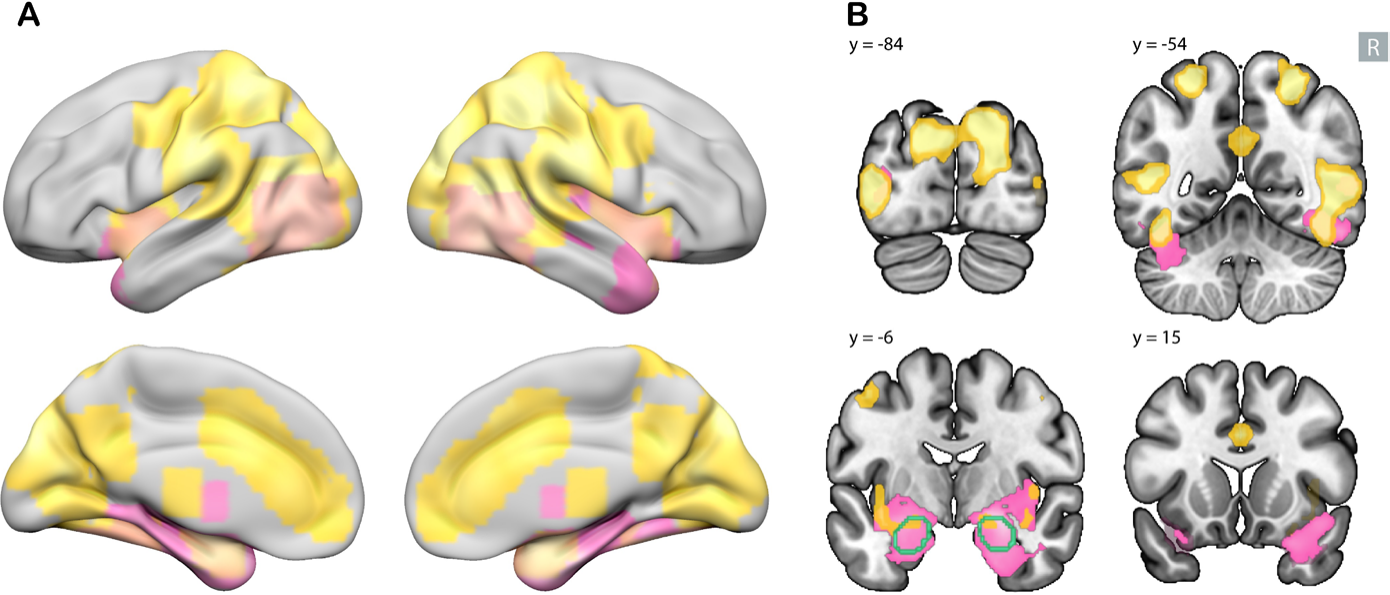
Clusters demonstrating a significant emotional memory enhancement with family-wise error (FWE)-corrected p < .05 and a minimum cluster size of five voxels. Yellow shows the results from the current study, while magenta depicts the results from a meta-analysis (using the Seed-based *d* Mapping (SDM) method with threshold-free cluster enhancement family-wise error rate *p* < .05) (Dahlgren et al., 2020), and salmon colour shows the overlap between the two. In panel (A), subcortical regions are projected to the surface. Panel (B) depicts regions in four coronal slices. Amygdala contours are shown in green. The R sign shows the right side of the brain.

To compare our results with the Dahlgren meta-analysis (Dahlgren et al., 2020), we used the emotional DM > neutral DM contrast. We identified large clusters in occipitotemporal, occipital, and anterior cingulate (ACC) cortices (Table 1; Fig. 3). Compared with the meta-analysis, both studies identified regions in the temporal–visual cortices, amygdala, and insula. However, we observed an extensive network in the temporoparietal region, anterior cingulate cortex, and posterior occipital cortex that was not reported in the meta-analysis, and contrary to the meta-analysis, we did not find any (para)hippocampal regions (Table 1; Fig. 3; Supplementary Figs. S5 and S6). To ensure comparability with the meta-analysis study, we also analysed data including the participants who did not show emotional memory enhancement (EEM) at the behavioural level. The key neural patterns and valence contrasts replicated the key conclusions of the EEM subgroup analysis (see Supplementary Fig. S7 and Table S1).

**Table 1.** Regions involved in emotional memory enhancement.

		Cluster		Peak				
Region	ROI (glass brain)	Size	P_FWE_	t	x	y	z	Network
Left lateral occipital ctx	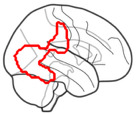	1318	<.001	13.9	-50	-74	4	VIS
Left fusiform ctx				8.5	-44	-47	-20	DATT
Left supramarginal ctx				7.8	-63	-28	24	SAL/VATT
Right middle temporal ctx	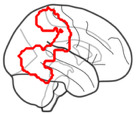	1689	<.001	12.2	52	-63	0	VIS
Right superior parietal ctx				8.9	30	-47	56	DATT
Right fusiform ctx				8.0	41	-52	-12	SAL/VATT
Right cerebral WM / superior parietal ctx	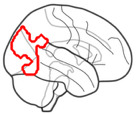	1052	<.001	8.5	16	-82	40	VIS
Right lateral occipital ctx				7.6	22	-82	20	
Left cerebral WM				7.3	-16	-85	32	
Left superior parietal ctx	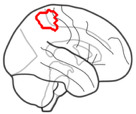	248	<.001	7.8	-30	-52	64	SomM DATT
Right insula	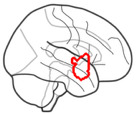	175	<.001	6.8	36	6	-16	SAL/VATT
Right insula				6.2	38	8	0	
Right insula				5.3	41	-6	0	
Left insula	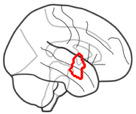	122	<.001	6.3	-33	-3	-16	SAL/VATT
Left insula				6.2	-38	-3	-8	
Left amygdala				5.6	-22	-3	-16	
Left caudal ACC	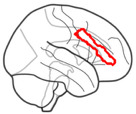	189	<.001	6.1	0	14	32	SAL/VATT
Right caudal ACC				5.8	3	30	16	Default
Right rostral ACC				6.1	3	38	8	Control
Right ventral DC	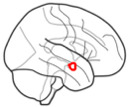	19	<.001	6.0	16	-3	-12	-
Left precentral ctx	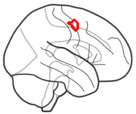	30	.001	5.9	-47	-3	56	DATT
Left cerebellum ctx-1	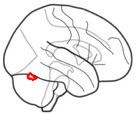	10	.002	5.1	-16	-72	-20	-
Left cerebellum ctx				4.8	-22	-63	-24	
Left cerebellum ctx-2	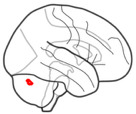	6	.005	5.0	-6	-72	-32	-
Right precentral ctx	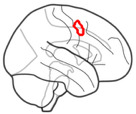	39	<.001	5.5	50	3	44	DATT
Left thalamus proper	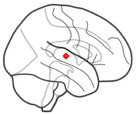	5	.007	5.0	0	-19	8	-

Clusters with a family-wise error (FWE)-corrected p-value <.05 and a minimum of five voxels, identified within the contrast of emotional subsequent memory (DM) > neutral DM. Regions are defined according to an in-house probabilistic atlas. Where clusters contain multiple peaks, secondary peaks are indicated in grey. In case the voxel with peak coordinates overlaps with white matter, the closest cortical region is mentioned as well. ACC: anterior cingulate cortex; DC: diencephalon. Network abbreviations include VIS: visual; SAL: salient; VATT: ventral attention; DATT: dorsal attention; SomM: somatomotor. WM: white matter; ctx: cortex.

### 3.3 Shared neural correlates of negative and positive emotional memory enhancement

**Fig. 4.**
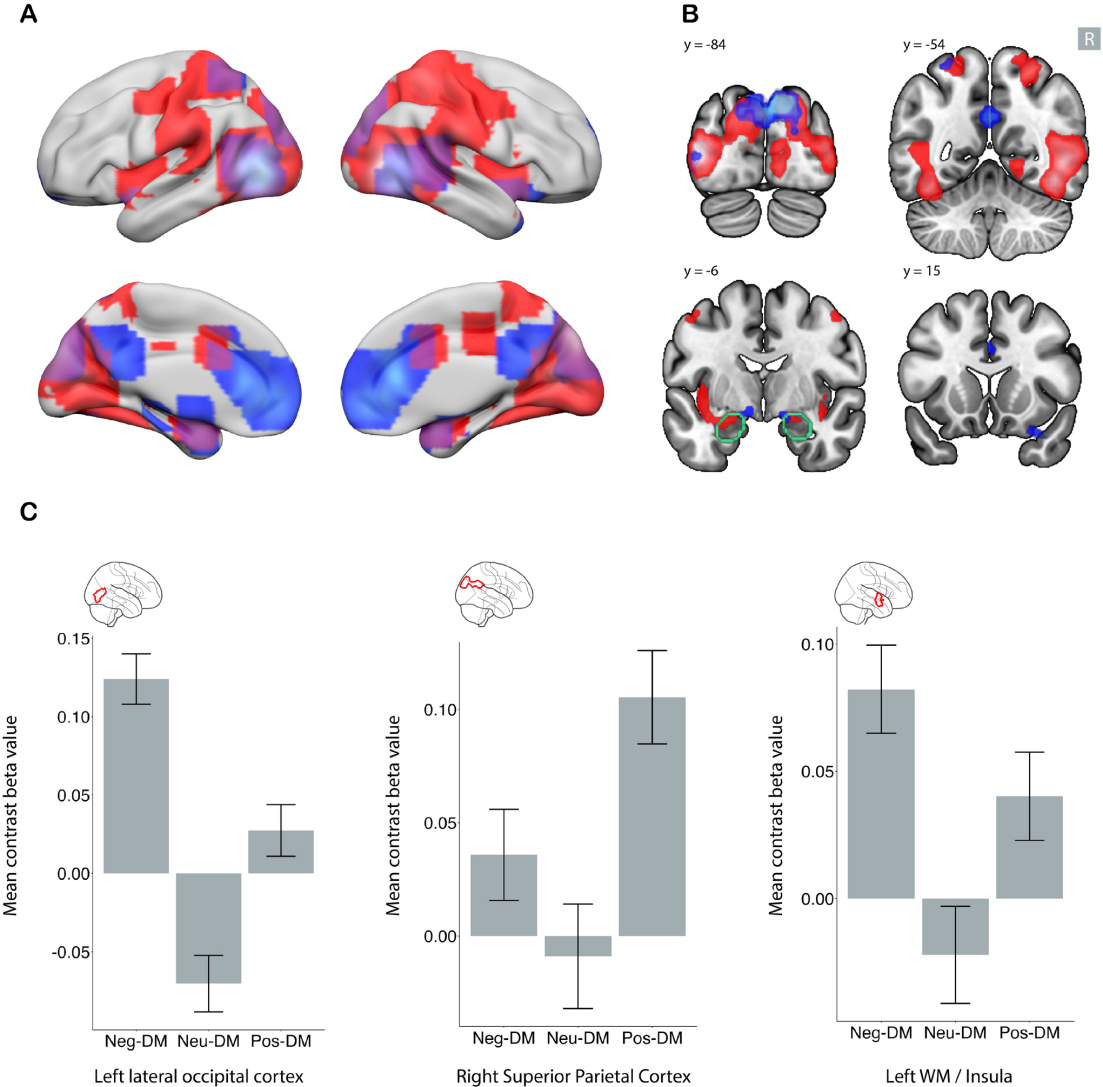
Shared and distinct brain regions in negative and positive emotional memory enhancement. Clusters demonstrating a significant association specifically with negative (red), specifically with positive (blue), and shared between positive and negative (purple in panel A) emotional memory enhancement. Whole-brain analysis with family-wise error (FWE)-corrected p < .05 and a minimum cluster size of five voxels. In panel (A), subcortical regions are projected to the surface. Panel (B) depicts the regions in four coronal slices. The R sign shows the right side of the brain, and the amygdala contour is depicted in green. Panel (C) shows the mean signal change for three sample clusters. In the whole-brain analysis, the left lateral occipital cortex showed significant signal for both the (negative DM > neutral DM) and (positive DM > neutral DM) contrasts. The left insula showed a greater signal specifically for the (negative DM > neutral DM) contrast, while the right superior parietal cortex showed a greater signal specifically for the (positive DM > neutral DM) contrast. Error bars represent the 95% confidence interval. DM: difference in memory contrast.

Regions shared between negative and positive emotional memory enhancement were identified by overlapping t-maps ((negative DM > neutral DM) ∩ (positive DM > neutral DM)), using an inclusive mask function in SPM. These regions include mostly occipital and superior parietal cortices within the visual network (Table 2, section 1; purple regions in Fig. 4).

**Table 2.** Shared and distinct regions for negative and positive emotional memory enhancement.

		Cluster		Peak				
Region	ROI (glass brain)	Size	P_FWE_	t	x	y	z	Network
**Section 1. (Neg DM > Neu DM) ∩ (Pos DM > Neu DM)**								
Left lateral occipital ctx	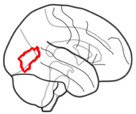	231	<.001	14.7	-50	-74	4	VIS
Right lateral occipital ctx-1	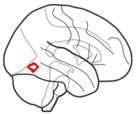	17	<.001	13.7	52	-66	-4	VIS
Right lateral occipital ctx-2	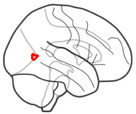	7	.004	13.9	52	-66	4	VIS
Right cerebral WM/superior parietal ctx	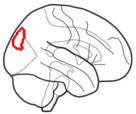	119	<.001	7.5	16	-82	40	VIS
Right cerebral WM-2/Right superior parietal ctx				6.9	22	-82	20	
Left cerebral WM/superior parietal ctx	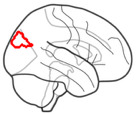	125	<.001	7.2	-8	-85	32	VIS
Left cuneus				6.0	0	-74	24	
Right BANKSSTS	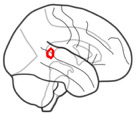	22	<.001	7.0	55	-41	16	SAL/VATT
Right cerebral WM/insula	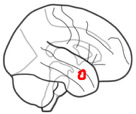	19	<.001	6.9	36	3	-16	SAL/VATT
Left cerebral WM/insula	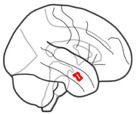	9	.002	6.6	-33	0	-20	SAL/VATT
Left superior parietal ctx	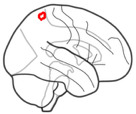	13	.001	5.8	-28	-52	64	DATT
**Section 2. Specific Neg DM > Neu DM**								
Right lateral occipital ctx	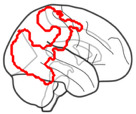	3924	<.001	14.2	52	-66	0	VIS
Left lateral occipital ctx				12.7	-47	-66	0	DATT
Left cerebral WM				12.2	-44	-82	8	
Left supramarginal ctx	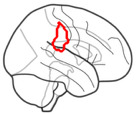	176	<.001	7.0	-63	-28	28	DATT
Left supramarginal ctx				6.8	-63	-25	36	SAL/VATT
Left cerebral WM				6.1	-52	-30	28	
Left cerebral WM/insula	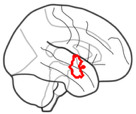	104	<.001	6.5	-33	0	-16	SAL/VATT
Left amygdala				6.0	-22	-6	-16	
Left insula				5.8	-38	-6	-12	
Right cerebral WM/insula	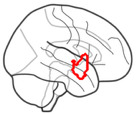	116	<.001	6.5	36	3	-20	SAL/VATT
Right insula				5.9	38	8	4	
Right insula				5.6	25	6	-20	
Left superior parietal ctx	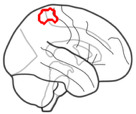	127	<.001	5.9	-22	-50	64	DATT
Left superior parietal ctx				5.3	-33	-50	64	
Left postcentral ctx				5.2	-41	-36	64	
Left precentral ctx	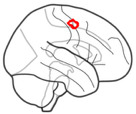	18	<.001	5.63	-50	-6	52	DATT
Left postcentral ctx				4.9	-44	-14	56	SomM
Left PCC	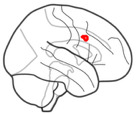	7	.004	5.1	0	8	36	SAL/VATT
Right PCC	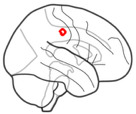	8	.003	5.0	6	-22	44	SAL/VATT
**Section 3. Specific Pos DM > Neu DM**								
Right superior parietal ctx	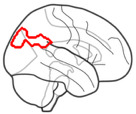	211	<.001	7.6	8	-85	40	VIS
Left cuneus				5.9	0	-88	28	Default
Left precuneus				5.7	0	-55	32	
Left rostral ACC	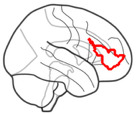	392	<.001	6.8	0	50	0	SAL/VATT
Right caudal ACC				6.8	0	30	16	Default
Left caudal ACC				5.2	0	16	32	Control
Left cerebral WM / ventral DC	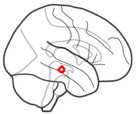	10	.002	5.4	-14	-25	-12	-
Left superior parietal ctx	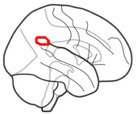	5	.007	5.3	-28	-58	68	DATT
Right superior temporal ctx	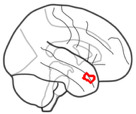	28	<.001	5.3	36	19	-28	Limbic
				4.9	30	8	-24	
Right ventral DC	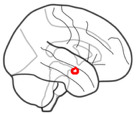	9	.003	5.2	14	-6	-12	-
Left cerebral WM/amygdala	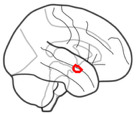	18	.001	5.1	-16	-6	-12	-
Left cerebral WM/temporal pole	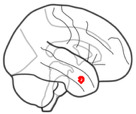	7	.004	5.1	-33	3	-28	-

Clusters demonstrating a significant association with negative/positive memory enhancement with family-wise error (FWE)-corrected p < .05 and a minimum cluster size of five voxels. This table presents (1) regions commonly activated in both negative DM > neutral DM and positive DM > neutral DM contrasts; (2) regions uniquely activated in negative DM > neutral DM (excluding regions also active in positive DM > neutral DM); and (3) regions uniquely activated in positive DM > neutral DM (excluding regions also active in negative DM > neutral DM). Anatomical locations are based on an in-house probabilistic atlas. Secondary peak coordinates within clusters are indicated in grey. In case the voxel with peak coordinates overlaps with white matter, the closest cortical region is mentioned as well. ACC, anterior cingulate cortex; PCC, posterior cingulate cortex; DC, diencephalon; BANKSSTS, banks of the superior temporal sulcus. Network abbreviations include VIS: visual; SAL: salient; VATT: ventral attention; DATT: dorsal attention; SomM: somatomotor; WM: white matter; ctx: cortex.

In ROI level analysis, all of the regions identified in this analysis showed deactivation for neutral DM contrast (Supplementary Figs. S8 and S9).

### 3.4 Neural correlates specific to negative emotional memory enhancement

Using the negative emotional memory enhancement map (negative DM > neutral DM) and masking out regions shared with positive emotional memory enhancement (positive DM > neutral DM), we identified areas specific to negative emotional memory enhancement (red areas in Fig. 4). These included the left superior parietal cortex, bilateral insula, and bilateral supramarginal cortex. Additionally, a large temporo-occipital region was detected; however, this area surrounded a region shared by both negative and positive valence (Table 2, section 2).

In ROI level analysis, all of these regions show either no activation or deactivation in neutral DM contrast. However, for the positive DM contrast, regions identified within the bilateral insula and right lateral occipital cortex showed more activity in the remembered condition than in the not-remembered condition (Supplementary Figs. S10 and S11).

### 3.5 Neural correlates specific to positive emotional memory enhancement

Using the positive emotional memory enhancement map (positive DM > neutral DM) and masking out regions shared with negative emotional memory enhancement (negative DM > neutral DM), we identified areas specific to positive emotional memory enhancement (blue areas in Fig. 4). These regions include the bilateral ACC, left precuneus, bilateral superior parietal cortices, and ventral DC (Table 2, section 3).

In ROI level analysis, most regions except the bilateral superior parietal cortex showed significant activity for negative DM and, to a lesser extent, for neutral DM contrasts, but the magnitude of activity for positive DM was higher than for the other two (Supplementary Figs. S12 and S13).

### 3.6 Neural correlates of negative and positive emotional memory enhancement, controlling for arousal

**Fig. 5.**
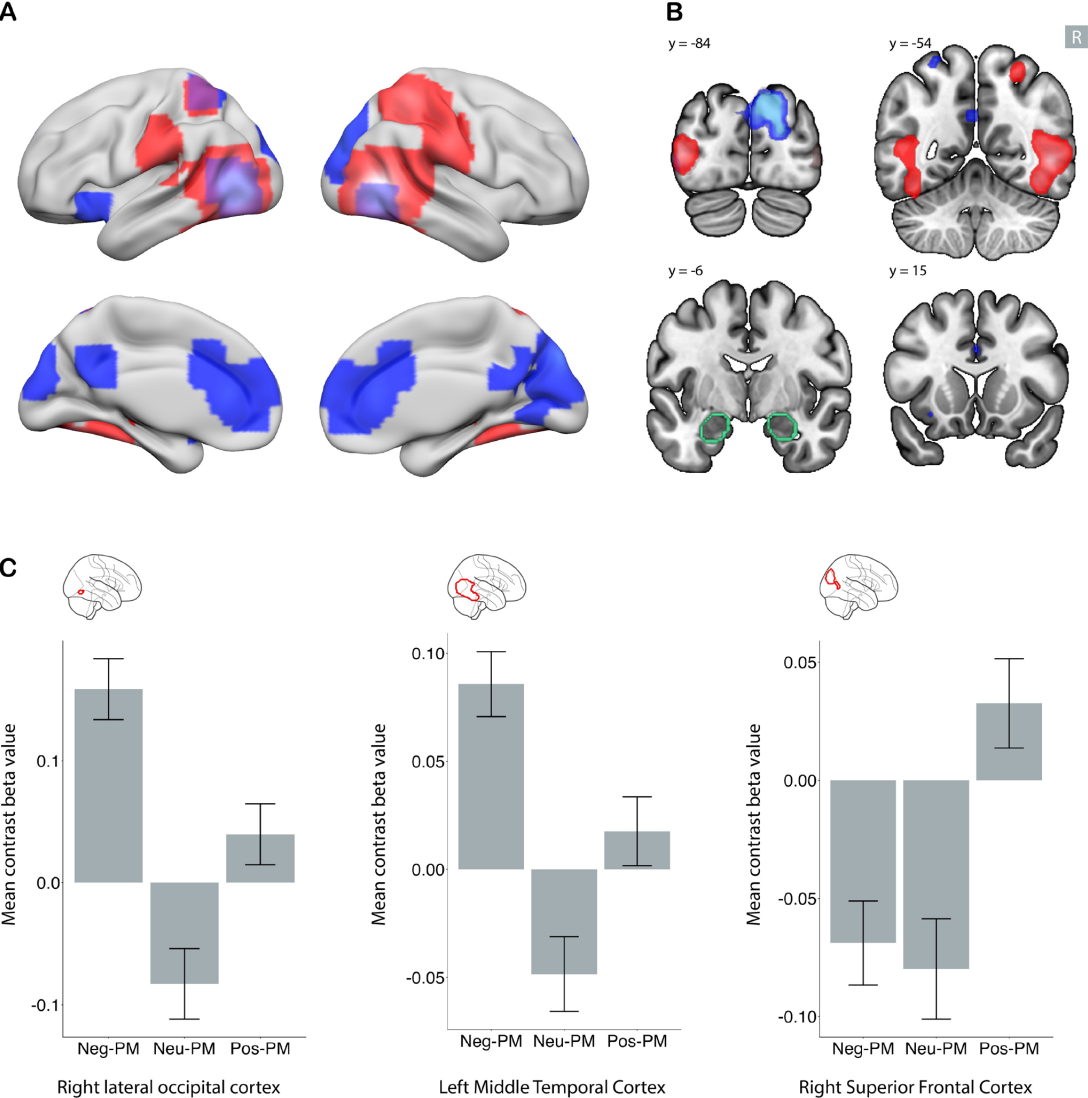
Shared and distinct brain regions in negative and positive emotional memory enhancement, after controlling for subjective arousal rating using parametric modulation. Clusters demonstrating a significant association specifically with negative (red), specifically with positive (blue), and shared between positive and negative (purple) emotional memory enhancement. Whole-brain analysis with family-wise error (FWE)-corrected p < .05 and a minimum cluster size of five voxels. In panel (A), clusters are projected to the surface. Panel (B) depicts the clusters in four coronal slices. The R sign shows the right side of the brain, and the amygdala contour is depicted in green lines. Panel (C) shows the mean signal change for three sample clusters. In the whole-brain analysis, after controlling for arousal, the right lateral occipital cortex was associated with both negative and positive memory parametric modulators (PM). The left middle temporal cortex was specifically associated with negative memory PM, while the right superior frontal cortex was specific to positive memory PM. Error bars represent the 95% confidence interval. PM: parametric modulator for memory.

When controlling for subjective arousal ratings using parametric modulation analysis, a reduction in the number of significant voxels was observed. Within the remaining clusters, two extensive clusters located in the left and right occipitotemporal cortices were identified as common to both negative and positive emotional memory enhancement. Notably, the cluster associated with negative emotional memory enhancement encompassed a greater number of voxels, resulting in a larger overall cluster size. However, the peak activation points for both negative and positive emotional memory enhancement contrasts demonstrated spatial overlap (Fig. 5; Table 3).

**Table 3.** Shared and distinct regions for negative and positive emotional memory enhancement controlled for subjective arousal rating using parametric modulation (PM).

		Cluster		Peak				
Region	ROI (glass brain)	Size	P_FWE_	t	x	y	z	Network
**Section 1. (Negative Memory PM > Neutral Memory PM) ∩ (Positive Memory PM > Neutral Memory PM)**								
Left lateral occipital ctx	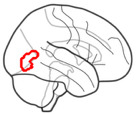	132	<.001	13.7	-50	-72	4	VIS
Right lateral occipital ctx	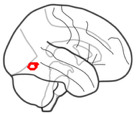	17	<.001	13.0	52	-66	-4	VIS
Left superior parietal ctx	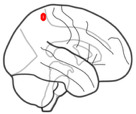	8	.003	5.1	-30	-50	64	DATT
**Section 2. Specific Negative Memory PM > Neutral Memory PM**								
Right lateral occipital ctx	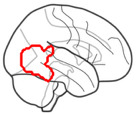	824	<.001	13.3	50	-63	0	VIS
Right cerebral WM				9.4	41	-50	-16	DATT
Right BANKSSTS				6.2	50	-41	16	SAL/VATT
Left middle temporal ctx	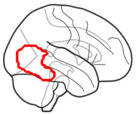	702	<.001	13.0	-50	-69	4	VIS
Left middle temporal ctx				11.0	-58	-72	4	DATT
Left lateral occipital ctx				12.1	-47	-77	8	
Right superior parietal ctx	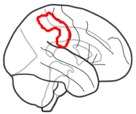	349	<.001	7.6	30	-47	56	SomM
Right supramarginal ctx				6.6	66	-19	40	DATT
Right cerebral WM				6.1	52	-22	32	
Left supramarginal ctx	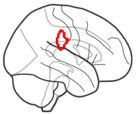	73	<.001	5.7	-63	-28	28	DATT
Left supramarginal ctx				5.6	-63	-25	36	SAL/VATT
Left postcentral cortex				4.8	-66	-16	32	
Left cerebral WM/ superior parietal ctx	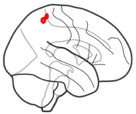	8	.003	4.9	-25	-52	60	DATT
Left superior parietal ctx				4.8	-33	-47	64	
**Section 3. Specific Positive Memory PM > Neutral Memory PM**								
Right superior parietal ctx	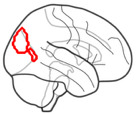	322	<.001	7.5	11	-85	40	VIS
Right cerebral white matter				6.3	22	-85	20	
Left cerebral white matter				5.2	-8	-88	32	
Left superior parietal ctx	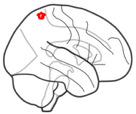	10	.002	6.0	-28	-52	68	DATT
Left caudal ACC-1	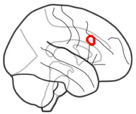	19	<.001	5.1	0	16	32	SAL/VATT Control
Left caudal ACC-2	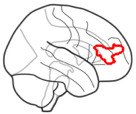	159	<.001	5.9	0	30	16	Default
Right rostral ACC				5.2	3	41	4	SAL/VATT
Right superior frontal ctx				5.2	3	60	16	Control
Left precuneus	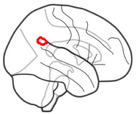	20	<.001	5.2	-3	-55	32	Default
Left lateral orbitofrontal ctx	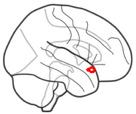	7	.004	5.1	-30	19	-12	Default

Clusters demonstrating a significant association with emotional memory enhancement with family-wise error (FWE)-corrected p < .05 and a minimum cluster size of five voxels after controlling for subjective arousal rating using parametric modulation (PM). This table presents (1) regions commonly activated in both negative > neutral and positive > neutral contrasts; (2) regions uniquely activated in negative > neutral (excluding regions also active in positive > neutral); and (3) regions uniquely activated in positive > neutral (excluding regions also active in negative > neutral). Anatomical locations are based on an in-house probabilistic atlas. Secondary peak coordinates within clusters are indicated in grey. In case the voxel with peak coordinates overlaps with white matter, the closest cortical region is mentioned as well. ACC, anterior cingulate cortex; BANKSSTS, banks of the superior temporal sulcus. Network abbreviations include VIS: visual; SAL: salient; VATT: ventral attention; DATT: dorsal attention; SomM: somatomotor; WM: white matter; ctx: cortex.

Beyond these shared regions, clusters within the bilateral supramarginal cortices, as well as lateral occipital and middle temporal regions, remained specifically associated with negative emotional memory enhancement. Conversely, clusters in the superior frontal cortex, superior parietal cortex, anterior cingulate cortex (ACC), and precuneus were specifically correlated with positive emotional memory enhancement (Fig. 5; Table 3).

After accounting for arousal, a large occipital cluster, and clusters in the insula and amygdala regions were no longer significantly implicated. This suggests that these regions play a role in the response to emotional arousal rather than being specific to emotional valence regarding emotional memory enhancement. In ROI level analysis, the bilateral lateral occipital cortices (shared between negative and positive memory enhancement) revealed significant activation for the negative memory regressor, significant deactivation for the neutral memory regressor, and low magnitude activation for the positive memory regressor (Supplementary Fig. S14). A similar pattern was evident in most regions identified as uniquely associated with negative memory enhancement, albeit with reduced activation magnitudes (Supplementary Fig. S15). In regions of the frontal lobe, ACC, and precuneus (predominantly specific to positive valence memory enhancement), we observed strong ROI activation for the positive memory regressor, with either no significant activation or activations of lower magnitudes for the neutral and negative memory regressors (Supplementary Fig. S16).

### 3.7 Neural correlates specific to neutral successful memory encoding

**Fig. 6.**
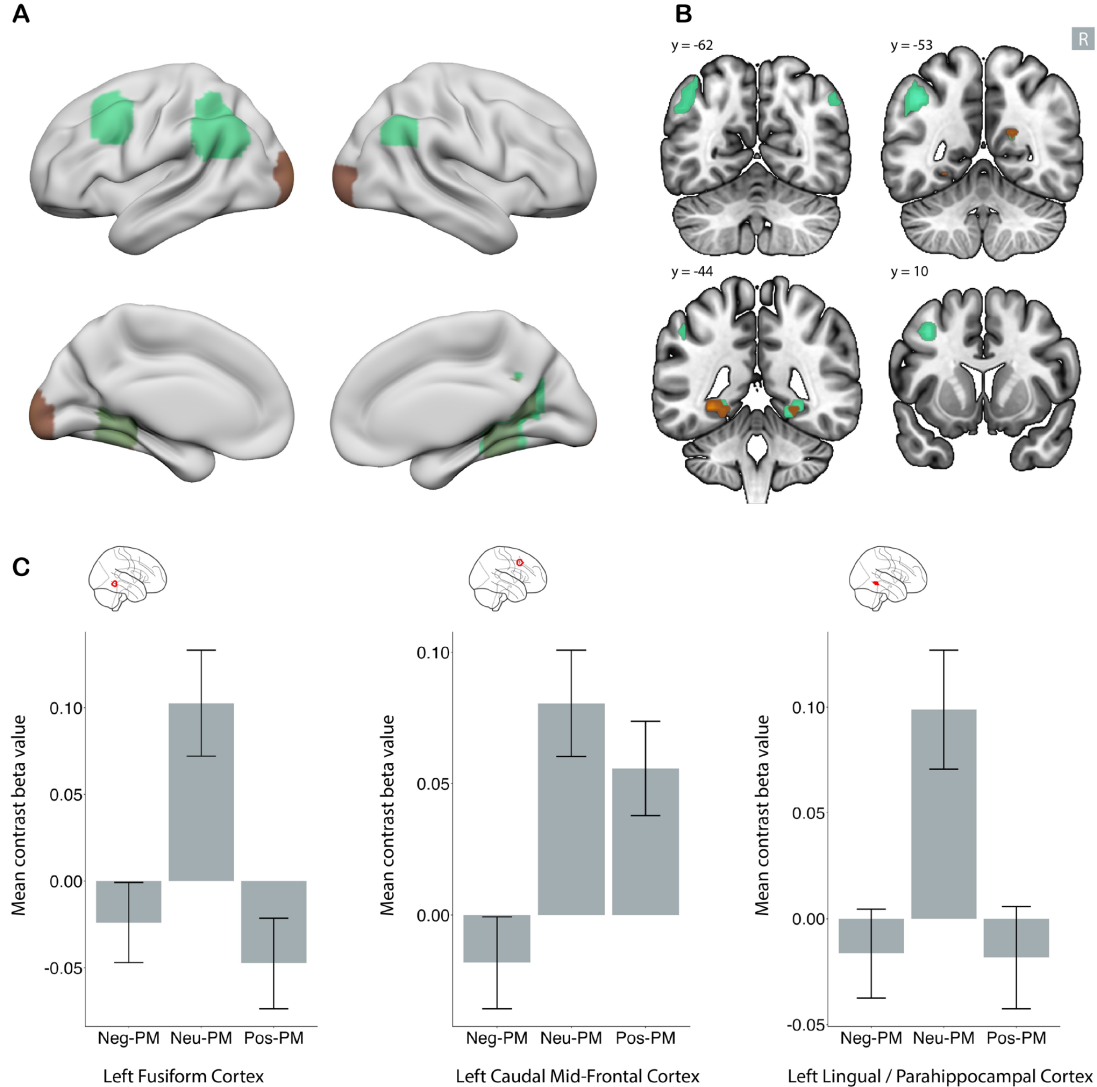
Brain regions showing higher activation in successful memory encoding in neutral compared with negative (green), or positive (brown), after controlling for subjective arousal rating. Whole-brain analysis with family-wise error (FWE)-corrected p < .05 and a minimum cluster size of five voxels. In panel (A), subcortical regions are projected to the surface. Panel (B) depicts the regions in four coronal slices. The R sign shows the right side of the brain. Panel (C) shows the mean signal change for three sample clusters. In the whole-brain analysis, after controlling for arousal, the right parahippocampal cortex and left fusiform showed greater signal for the neutral memory parametric modulator (PM) than both the negative and positive PMs. The left caudal mid-frontal cortex showed greater signal for the neutral PM compared with the positive PM. Error bars represent the 95% confidence interval. PM: parametric modulator for memory.

Following parametric modulation analysis to control for arousal, which is crucial when comparing neutral with emotional encoding, we detected clusters in fusiform, precuneus, and parahippocampal cortices, which showed more activation for the neutral memory modulator than for the emotional ones. Multiple clusters in the frontal and parietal regions and two clusters in the bilateral lingual/parahippocampal region were identified as having higher activation in the neutral memory modulator than in the negative memory modulator. Moreover, a cluster within the left lateral occipital cortex displayed increased activation for the neutral memory modulator compared with positive memory modulators (Fig. 6; Table 4). ROI analysis confirmed these findings, demonstrating a positive association with neutral memory-PM in these regions (Supplementary Figs. S17–S19). The same analysis without controlling for arousal was performed, which was, in most parts, in line with the arousal-controlled analysis (Supplementary Figs. S20–S24 and Table S2).

**Table 4.** Shared and distinct regions for neutral successful memory encoding compared with positive and negative successful memory encoding controlled for subjective arousal rating using parametric modulation (PM).

		Cluster		Peak				
Region	ROI (glass brain)	Size	P_FWE_	t	x	y	z	Network
**Section 1. (Neutral Memory PM > Negative Memory PM) ∩ (Neutral Memory PM > Positive Memory PM)**								
Left fusiform	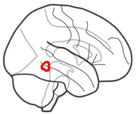	27	<.001	5.8	-30	-44	-8	VIS
Right parahippocampal ctx	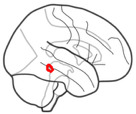	13	.001	5.4	25	-41	-12	VIS
Right precuneus ctx	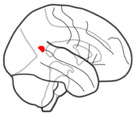	5	.007	5.4	19	-52	20	Default
**Section 2. Neutral Memory PM > Negative Memory PM**								
Left cerebral WM / caudal middle frontal ctx	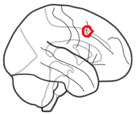	57	<.001	6.0	-33	14	44	Default
Right parahippocampal ctx	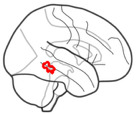	26	<.001	6.0	19	-41	-12	VIS
Right lingual ctx				5.9	28	-47	-8	
Left cerebral WM/inferior parietal ctx	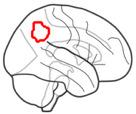	152	<.001	5.8	-44	-52	40	Control
Left inferior parietal				5.3	-50	-60	36	Default
Right precuneus ctx	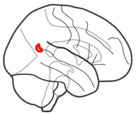	11	.002	5.7	19	-58	20	Default
Left lingual/parahippocampal ctx	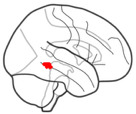	7	.004	5.1	-22	-47	-4	VIS
Right inferior parietal ctx	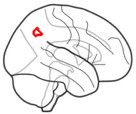	15	.001	5.0	52	-63	40	Control
**Section 3. Neutral Memory PM > Positive Memory PM**
Left lateral occipital ctx	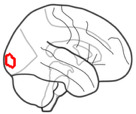	77	<.001	6.9	-19	-102	4	VIS
Left cerebral WM/fusiform	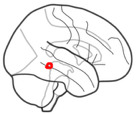	8	.003	5.9	-33	-41	-8	VIS
Right cerebral WM/lateral occipital ctx	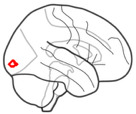	13	.001	5.2	25	-94	-4	VIS

Clusters demonstrating a significantly higher association with neutral successful memory encoding than negative or positive successful memory encoding after controlling for arousal (family-wise error (FWE)-corrected p < .05 and a minimum cluster size of five voxels) using parametric modulation (PM). This table presents (1) regions activated in both neutral > negative and neutral > positive, (2) regions uniquely activated in neutral > negative (excluding regions also active in neutral > positive), and (3) regions uniquely activated in neutral > positive (excluding regions also active in neutral > negative). Anatomical locations are based on an in-house probabilistic atlas. Secondary peak coordinates within clusters are indicated in grey. In case the voxel with peak coordinates overlaps with white matter, the closest cortical region is mentioned as well. Networks abbreviations include VIS: visual. WM: white matter; ctx: cortex.

## 4 Discussion

In this large-scale fMRI study (N = 1006), we provide several novel insights into the neural underpinnings of emotional memory enhancement. When comparing brain activity for emotional DM versus neutral DM, in contrast to the findings reported in the meta-analysis (Dahlgren et al., 2020), we did not observe (para)hippocampal involvement. However, we uncovered a previously underreported temporoparietal network, particularly involving the supramarginal gyrus and superior parietal lobule. When controlling for subjective arousal, we found that the insula and amygdala were no longer related to emotional memory enhancement, suggesting that these regions play a role in the response to emotional arousal rather than being specific to emotional valence. After controlling for arousal, we also dissociated valence-specific regions, such as the lateral occipital cortices for negative stimuli, and control- and default-mode networks-related activations for positive memory encoding. Additionally, we found that successful neutral memory encoding uniquely involved frontoparietal control networks.

### 4.1 Behavioural findings

At the behavioural level, we observed a clear priority in remembering emotional stimuli over neutral ones. Moreover, stimuli with positive emotional valence were remembered more frequently than those with negative valence. Notably, negative stimuli were rated as more arousing than positive ones, a factor crucial for interpreting neural activity associated with valence and underscoring the necessity of controlling for arousal in the analyses.

The superior recall of positive relative to negative stimuli was somewhat unexpected, given that negative images were associated with higher subjective arousal. However, the magnitude of this effect was small (|Cohen’s d| = 0.25). While the valence groups were matched for low-level visual features and content, other dimensions known to influence memorability, such as social and functional content, semantic richness, or conceptual distinctiveness, were not explicitly controlled (Khosla et al., 2015; Rust & Mehrpour, 2020). Further, because memory was assessed using a free-recall test, performance may depend on stimulus nameability (i.e., how easily participants can generate a brief verbal description). Together, arousal alone cannot fully explain emotional memory performance, and additional stimulus- and retrieval-dependent factors likely contribute to the observed positive memory advantage.

### 4.2 Emotional memory enhancement

In line with the latest meta-analysis (Dahlgren et al., 2020), we identified the amygdala, insula, and bilateral visual cortex as regions involved in emotional memory enhancement (Fig. 3). Moreover, we identified regions in the anterior cingulate cortex (ACC) and a broader temporoparietal cluster extending to the supramarginal gyrus, which was not found in the meta-analysis but reported in individual studies (Kensinger & Schacter, 2008; Murty et al., 2010; Ritchey et al., 2011). In contrast, Dahlgren et al. (2020) reported substantial involvement of regions in the hippocampus and parahippocampal region that were not identified in our study. One important factor to consider in comparing our study with the meta-analysis study is the task paradigms used in each. There are two main differences to note: First, in our study, we only used pictures as stimuli, whereas the meta-analysis reviews studies with both words and pictures. Second, to categorise pictures as remembered and not remembered, we relied on a free recall task, while most of the studies included in the meta-analysis used a recognition paradigm. A closer look at studies included in the meta-analysis (Dahlgren et al., 2020) that used the free recall paradigm and emotional memory enhancement contrasts shows that only one study reported a small parahippocampal cluster, and the others did not report any (para)hippocampal regions (Barnacle et al., 2016; Etkin et al., 2011; Rasch et al., 2009). In contrast, in studies which used recognition, these regions were reported repeatedly (Mickley Steinmetz & Kensinger, 2009; Mickley Steinmetz et al., 2012).

Recognition and recall differ not only in task demands but also in the memory processes they engage. Recognition performance can be supported by familiarity-based mechanisms or low-confidence memory signals, whereas free recall places greater demands on self-initiated retrieval and episodic recollection-based processes (Squire et al., 2007; Tulving, 2002; Yonelinas et al., 2024). Prior recognition-memory studies have demonstrated that neural subsequent memory effects differ depending on whether memory is supported by recollection versus familiarity, and on the confidence of recognition responses (Yonelinas et al., 2010). For example, high-confidence/recollection-based recognition has been linked to stronger engagement of the bilateral hippocampus and parahippocampal cortex regions than low-confidence/familiarity-based recognition (Kim, 2021). Importantly, the forgotten category is also defined differently: items that are not recalled may nevertheless be recognised (often at lower confidence), meaning that recall-based forgotten trials can still include weaker memory traces. This can reduce the contrast between remembered and forgotten trials in medial temporal regions and may help explain why (para)hippocampal involvement was less evident in our recall-based study compared with recognition-heavy literatures. Despite these paradigm differences, our results largely converge with the overall pattern reported in the meta-analysis (Dahlgren et al., 2020).

Notably, after controlling for arousal, which was not applied in the meta-analysis, clusters in the cerebellum, amygdala, and two substantial insular clusters were no longer implicated. This suggests that these regions play a role in the response to emotional arousal rather than being specific to emotional valence in enhancing memory. Our findings align with previous reports on amygdala activity in response to emotional arousal (Adolphs et al., 1999; Cahill & McGaugh, 1995; Canli et al., 2000) and Kensinger’s proposed theory of an amygdala–hippocampal pathway, which is more closely related to arousal aspects of emotional memory enhancement (Kensinger & Corkin, 2004).

To clarify whether the identified DM effects reflect regions specific to memory success or those tracking stimulus properties regardless of memory outcome, we inspected the ROI level beta estimates for forgotten items (Supplementary Fig. S5). We observed that most of the regions exhibited higher activation for both remembered and forgotten emotional items than for neutral items, however, this difference was larger in some regions including insula/amygdala cluster and caudal anterior cingulate cortex. This pattern is consistent with an effect of degree, whereby these regions are sensitive to arousal or salience of the stimuli automatically, though their engagement is significantly intensified during successful encoding (Supplementary Fig. S6).

### 4.3 Shared brain regions between negative and positive emotional memory enhancement

Among the regions associated with both negative and positive emotional memory enhancement are sensory areas, including the lateral occipital and superior parietal cortices. Additionally, we identified a cluster in the banks of the superior temporal sulcus. When compared with resting-state networks, these regions overlap with a large visual network and a salience-attention network.

However, after controlling for arousal, only two clusters located in the left and right occipitotemporal cortices were identified as common to both negative and positive emotional memory enhancement. At the ROI level, we observed that the lateral occipital regions were primarily associated with negative DM. Their presence in positive DM > neutral DM contrast appears to result from a negative association between neutral DM and activity in these regions (Supplementary Fig. S9). However, inspection of the beta values at the ROI level (Supplementary Fig. S8) reveals that these regions show greater activation during emotional events than during non-emotional ones. This suggests that while these visual cortex regions are involved in emotion recognition, their contribution to successful memory encoding is specific to negative valence. Notably, these are the two main clusters that remained significant after controlling for arousal, indicating that their association with negative DM can be attributed to emotional valence.

### 4.4 Neural correlates specific to negative emotional memory enhancement

Regions implicated exclusively in negative emotional memory enhancement include a large cluster covering the lateral occipital, fusiform gyrus, pericalcarine, and inferior temporal cortices bilaterally. Of note, this large cluster also overlaps with shared regions in bilateral occipital cortices discussed earlier, and substantial parts of these clusters survived after controlling for subjective arousal ratings, indicating their primary role in valence-dependent negative emotional memory enhancement. Importantly, clusters in bilateral supramarginal gyri and the fusiform gyrus survived after controlling for arousal.

Previous studies have shown that the supramarginal gyrus, in association with the anterior insula, plays a role in empathy feelings through self-other distinction (Hoffmann et al., 2016; Silani et al., 2013; Zhao et al., 2021). In another study, focusing on atrophied brain regions, the supramarginal gyrus grey matter volume was correlated with emotion recognition performance (Wada et al., 2021). Although none of these studies remarks on the supramarginal gyrus’s function specificity for negative stimuli, most of them focused on negative stimuli (such as pain) in the tasks.

The fusiform gyrus is well established as a key region involved in high-level visual processing, facial emotion recognition, and the evaluation of emotional intensity (Jung et al., 2021; Kanwisher & Yovel, 2006; Zhang et al., 2016). It also interacts closely with subcortical structures such as the amygdala and hippocampus, emphasising its role in emotional memory formation (Fenker et al., 2005; Frank et al., 2019; Vuilleumier et al., 2004). In our findings, we observed overlapping activation within the fusiform gyrus for both negative and positive DM, with a notably larger cluster for negative stimuli. This observation is further supported by our ROI analyses and is consistent with previous research on emotional memory (Kark & Kensinger, 2015; Kensinger & Schacter, 2008; Mickley & Kensinger, 2008). The involvement of the fusiform gyrus alongside the supramarginal gyri and insula may point to a broader network underlying empathic processes.

### 4.5 Neural correlates specific to positive emotional memory enhancement

Brain regions associated exclusively with positive emotional memory enhancement were mostly distributed around the midline, including the superior frontal, anterior cingulate, and precuneus cortices, plus a superior parietal cluster that extends to the primary visual cortex, overlapping bilateral cuneus. The activity of these regions was independent of the arousal effect and formed a pattern resembling the default mode network, indicating the importance of this network in positive emotional memory enhancement. Among these regions, the cuneus, precuneus, and frontal cortices have been repeatedly reported in previous studies (Botzung et al., 2010; Dolcos et al., 2012; Kensinger & Schacter, 2008; Mickley Steinmetz & Kensinger, 2009; Ritchey et al., 2011) to be associated with positive emotional memory enhancement. The involvement of higher cognitive regions in this network has been interpreted as more semantically controlled strategies for successful positive memory encoding (Dolcos et al., 2017). These findings partly align with Kensinger’s proposed second route for memory, comprising a frontal–hippocampal network (Kensinger & Corkin, 2004).

However, valence-specific activity for ACC was only observed in one study and only in a subgroup of older participants (Kensinger & Schacter, 2008). ACC, in collaboration with the orbitofrontal cortex, has been associated with reward perception and top–down emotion regulation (Etkin et al., 2011; Rolls, 2019). In our study, the rostral and caudal regions of the ACC were associated exclusively with positive DM. This observation is in line with experiments showing ventral regions of the anterior cingulate cortex as more responsive to pleasant experiences (Grabenhorst & Rolls, 2011; Rolls, 2019). These clusters survive after controlling for subjective arousal, showing an arousal-independent effect in successful positive memory encoding. A lesion study has also shown that patients whose rostral ACC has been surgically removed show difficulties in face and voice emotional expression identification, and experience changes in the subjective emotional state, whereas those with lesions in more posterior parts did not show such changes (Hornak et al., 2003). These observations indicate an important arousal-independent role of ACC in emotion recognition and regulation that contributes to the successful encoding of positively valenced stimuli.

### 4.6 Neural correlates specific to successful memory encoding of neutral items

In the recent meta-analysis, it was suggested that there is a need to examine the so-called deactivation of brain regions in emotional memory enhancement contrasts, as these deactivations could play a role at the network level for successful memory encoding (Dahlgren et al., 2020). However, it could also be the case that for remembering neutral events, different strategies are engaged, and higher activation for neutral DM than for emotional DM cannot necessarily be related to deactivation in the emotional memory enhancement mechanisms. It should be noted that neutral pictures were rated as low-level arousing, and therefore, we focus on the results after controlling for the arousal effect.

Our analysis revealed that, compared with negative and positive DM, neutral DM was associated with increased activity in clusters within the visual cortex. These regions align with the occipital–inferior temporal pathway, which is implicated in object recognition (Grill-Spector & Malach, 2004). Notably, we also observed parahippocampal/lingual activation that was specific to the neutral DM in contrast to the negative (bilaterally) and positive (unilaterally) DM. This finding contrasts with Dahlgren’s meta-analysis (Dahlgren et al., 2020), which linked parahippocampal activity to emotional memory enhancement. The identified regions overlap with the parahippocampal place area and are part of the ventral visual stream, known for its role in encoding scenes and places (Epstein et al., 1999). It is worth noting that while the picture set was controlled for the number of objects, human figures, and scenes across the three valence categories, this does not guarantee that the same types of stimuli were equally remembered. Therefore, if neutral images containing such objects were more frequently remembered than other neutral or emotional stimuli, the observed activation would be expected. To assess whether such differences could systematically bias the results, we conducted a trial-level logistic mixed-effects analysis with subject- and item-level random intercepts. This analysis showed no evidence that the relationship between content category and memory differed across valence categories (i.e., no valence × content interaction; F = 0.84, p > .05), suggesting that any item-to-item variability in memorability was not selectively driven by specific content types within a given valence condition. Supplementary Figure S2 shows a descriptive visualisation of the average probability of recall by content and valence, but the inferential conclusion is based on the item-level mixed-effects model, which appropriately accounts for both subject and item variability.

Moreover, in comparison with the negative DM, the neutral DM was associated with several clusters involved in the default mode and control networks. This may reflect the increased cognitive elaboration involved in encoding emotionally neutral information, which lacks the automatic salience typically associated with negative content.

### 4.7 Theoretical Implications: Refining Models of Valence-Specific Memory Enhancement

The present findings help clarify two uses of the term “valence effects” in the emotional-memory literature: (i) effects of valence under relatively low arousal (“valence-only”), often linked to more controlled/elaborative encoding processes, and (ii) effects of valence when stimuli are emotionally arousing but differ in valence (e.g., negative vs positive arousing), which may bias processing routes (Kensinger & Kark, 2018).

When subjective arousal was not explicitly modelled, we observed engagement of the amygdala and insula, consistent with accounts in which arousal recruits an amygdala-mediated alerting/salience system that prioritises emotional content (Pessoa & Adolphs, 2010; Pourtois et al., 2013). When statistically accounting for subjective arousal using parametric modulation, valence-specific effects remained: negative successful encoding preferentially involved temporo-occipital sensory regions (e.g., fusiform gyrus, lateral occipital cortex), whereas positive successful encoding preferentially engaged midline and prefrontal networks.

This pattern is consistent with the idea that, beyond arousal, valence can bias the neural systems that support successful encoding. In particular, the stronger involvement of high-level visual regions for negative items aligns with sensory-tuning accounts of negative valence (e.g., “Negative Emotional Valence Enhances Recapitulation” (NEVER) model, Bowen et al., 2018), although the NEVER model’s emphasis on retrieval recapitulation was not directly tested here. Likewise, greater engagement of midline/prefrontal networks for positive items may reflect relatively greater involvement of higher-order elaborative/semantic processes. Furthermore, these dissociations may also be interpreted in terms of underlying motivational states as suggested by Clewett and Murty (2019). In this framework, negative stimuli may engage an arousal-related “narrowing” mechanism that prioritises item-specific sensory features, whereas positive stimuli may promote a behavioural-activation-related broadening that supports more integrative and schematic representations. Together, these findings suggest that valence may contribute, independently of subjective arousal, to qualitative differences in the encoding pathways that support later remembering.

### 4.8 Strengths and limitations

A key strength of the present study lies in using a large-scale fMRI sample, which helps to overcome issues of low statistical power that often limit the generalisability of neuroimaging findings. This allows for more reliable inferences about brain–behaviour relationships involved in emotional memory enhancement.

However, several limitations should be considered when interpreting the results. (1) Subjective arousal ratings may not fully align with physiological arousal. Future studies should incorporate physiological measures such as skin conductance. (2) Our rating scales were coarse (three-point scales), limiting nuanced interpretation. (3) While arousal could be modelled as a parametric modulator due to its ordinal structure and within-category variability, valence was coded as a categorical variable with no meaningful linear ordering. It was, therefore, not possible to investigate arousal effects on memory while controlling for valence in parametric modulation. (4) In the current study, valence was defined using normative rather than individual subjective ratings. While this approach ensured consistency across participants and control over low-level visual features, it may have overlooked subtle individual differences in emotional perception. (5) Although the large sample size provided substantial between-subject statistical power, subsequent memory analyses are inherently constrained by the number of remembered trials per participant and condition. We, therefore, applied an inclusion criterion requiring a minimum number of remembered events per valence category to reduce extreme sparsity and improve the estimatability of first-level contrasts. However, because remembered and forgotten trials are typically unbalanced within individuals, the precision of within-subject subsequent-memory estimates may remain lower for participants with fewer remembered trials.

### 4.9 Conclusion

This large-scale fMRI study provides robust evidence for distinct neural underpinnings of emotional memory enhancement. By controlling for subjective arousal, we identified both valence-independent and valence-specific brain regions. Our findings indicate that the insula and amygdala are primarily involved in arousal-related processes, while negative and positive emotional memories engage partially overlapping but also distinct networks—sensory-attentional regions for negative stimuli and default-mode regions for positive ones. In contrast, neutral memory relies more on scene-related and frontoparietal control regions. These results refine current models of emotional memory and underscore the importance of jointly considering valence and arousal in future research.

## Supplementary Material

Supplementary Material

## Data Availability

Participant-level data and analysis code are hosted in controlled-access repositories at the University of Basel and will be shared upon reasonable request to the corresponding author, subject to a data-sharing agreement and institutional approval. Any shared datasets will be de-identified in line with applicable regulations. The group-level results of the current study, including neuroimaging nifti files, can be accessed through the Neurovault repository (https://neurovault.org/collections/JOAVVSYP/).
