# Supplementary material for: Neural correlates of emotional memory enhancement: The role of valence and arousal
Source: Imaging Neurosci (Camb). 2026 Apr 17;4:IMAG.a.1213. doi: 10.1162/IMAG.a.1213 (PMC13094029; doi:10.1162/IMAG.a.1213)
Supplement: Supplementary Material [file IMAG.a.1213_supp.pdf]

Neural Correlates of Emotional Memory Enhancement: The Role of Valence and Arousal
(Supplementary Material)

Manuscript Nr: IMAG-25-0742

Authors:

Ehssan Amini[1,2], David Coynel[1,2], Andreas Papassotiropoulos[2,3,4], Dominique J.-F. de Quervain[1,2,3]

Affiliations:

[1] Division of Cognitive Neuroscience, Department of Biomedicine, University of Basel, CH-4055 Basel, Switzerland
[2] Research Cluster Molecular and Cognitive Neurosciences, Department of Biomedicine, University of Basel, CH-4055 Basel, Switzerland
[3] Psychiatric University Clinics, University of Basel, CH-4055 Basel, Switzerland
[4] Division of Molecular Neuroscience, Department of Biomedicine, University of Basel, CH-4055 Basel, Switzerland

Corresponding authors:

Ehssan Amini: ehssan.amini@unibas.ch; Dominique de Quervain: dominique.dequervain@unibas.ch

ORCID: https://orcid.org/0000-0001-7131-2464

Supplementary Material

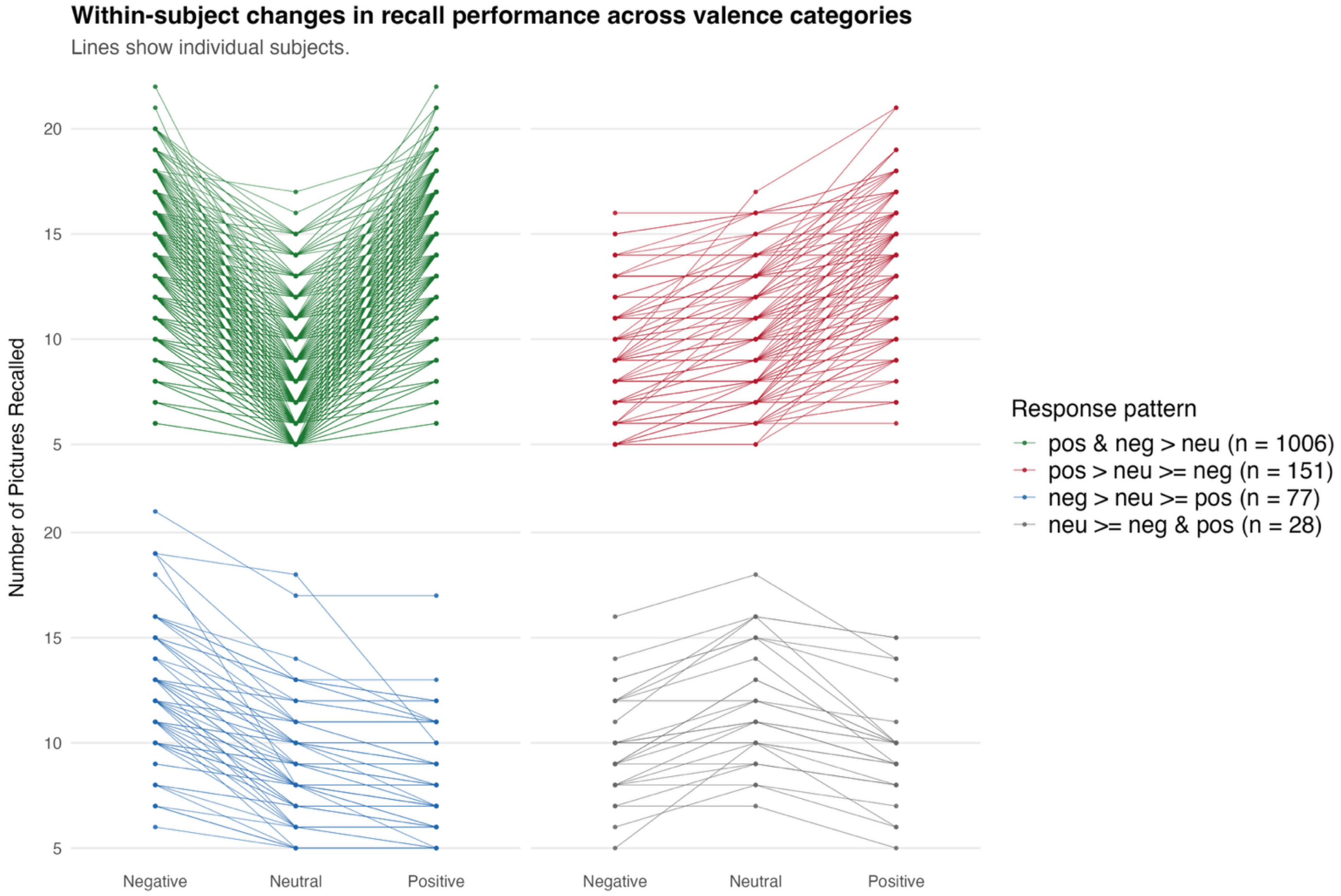

Supplementary Figure 1. Within subject free recall memory performance across valence categories for the subjects who remembered at least 5 pictures per category. Each dot/line shows one participant. Y axis shows the number of pictures remembered and X axis shows the valence category. The subjects who showed higher performance for both negative and positive compared to neutral were used for fMRI analysis (Pos & neg > neu (N = 1006)).

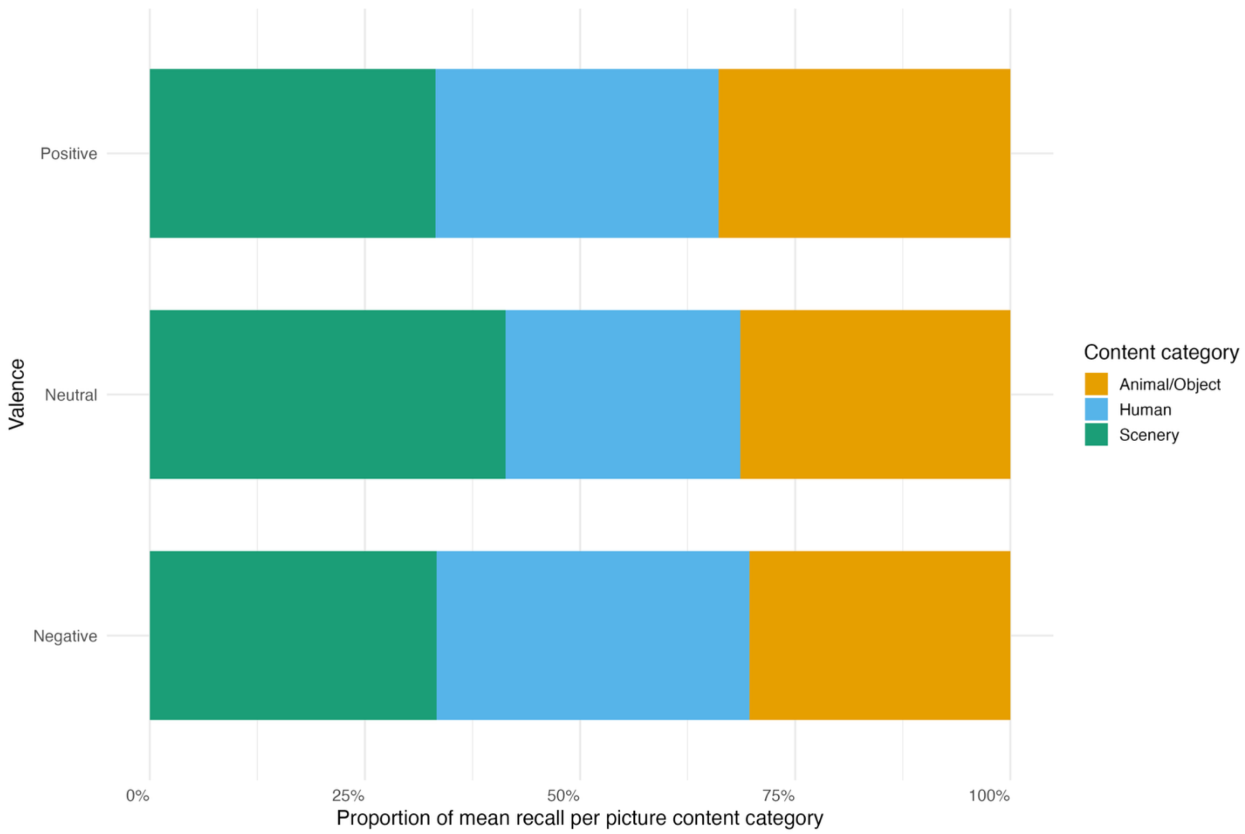

Supplementary Figure 2. Comparison of proportional memorability across picture categories within each valence. Proportions were calculated by dividing the mean number of times pictures within each category were recalled by the mean total number of recalled pictures across all categories.

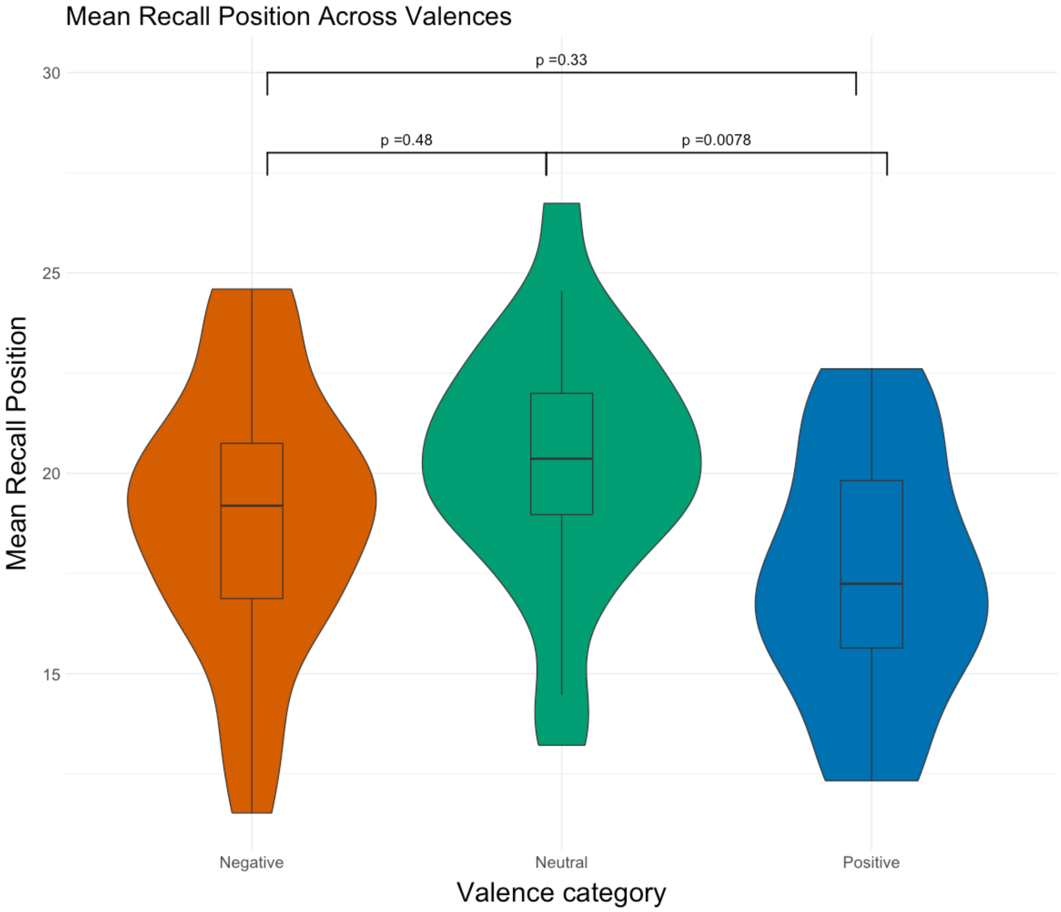

Supplementary Figure 3. Recall output order by valence. Distribution of mean recall position across images within each valence category. Emotional images were recalled earlier than neutral images, with no significant difference between positive and negative images. P values are derived from pairwise Student's t-test and Bonferroni corrected.

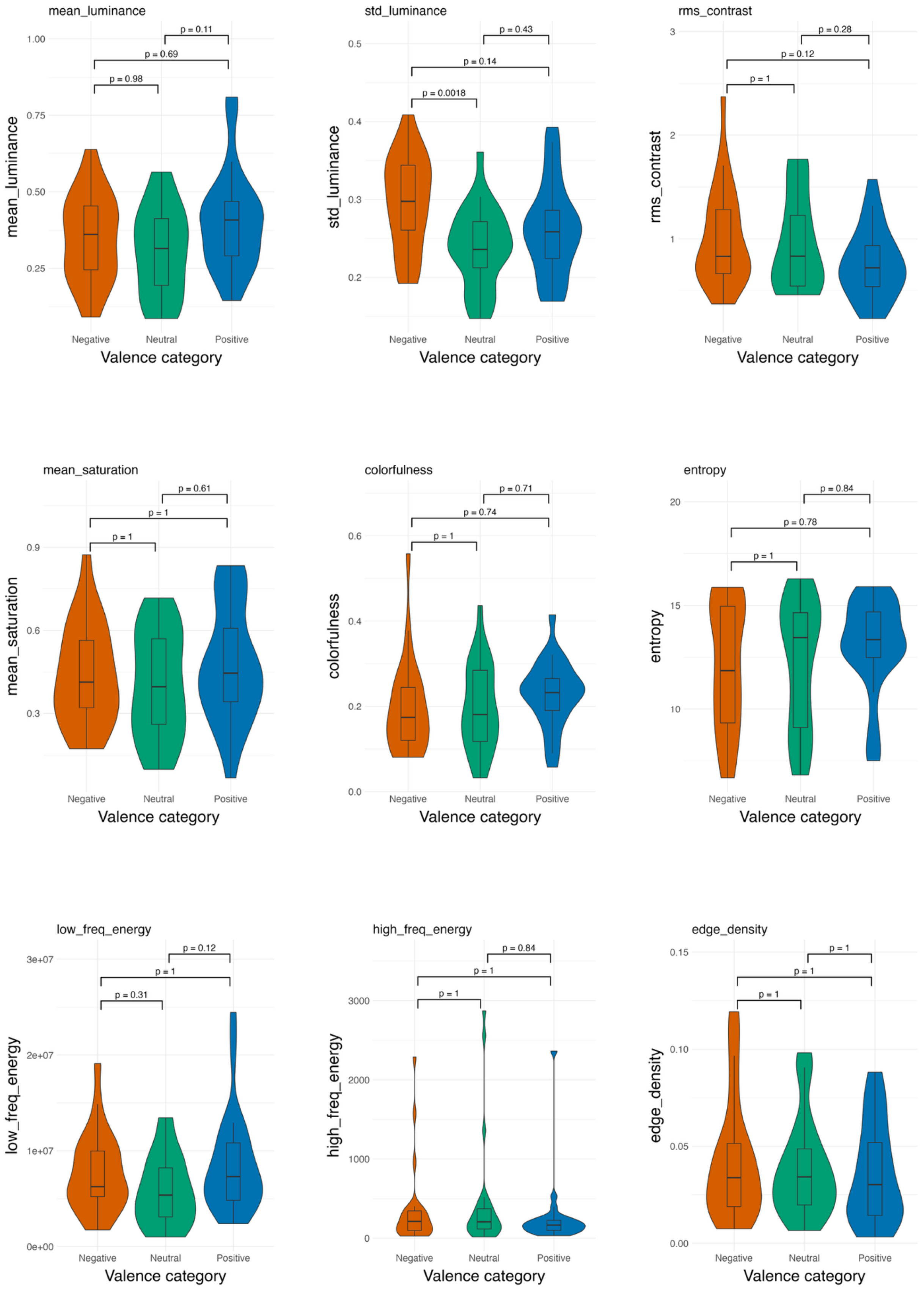

Supplementary Figure 4. Panels show comparison of mean low-level visual features extracted from each valence category. Mean luminance reflects the average image brightness, while standard deviation of luminance (std_luminance) captures variability in brightness across pixels. RMS contrast quantifies overall contrast strength in the image. Mean saturation represents average color intensity, and colorfulness captures the diversity and vividness of colors. Entropy measures the statistical complexity of pixel intensity distributions, with higher values indicating greater visual information content. Low-frequency energy reflects coarse, global image structure, whereas high-frequency energy indexes fine-grained details and texture. Edge density reflects the proportion of edge pixels and indexes the amount of contour information in the image. P values are derived from pairwise Student's t-test and Bonferroni corrected for each feature separately.

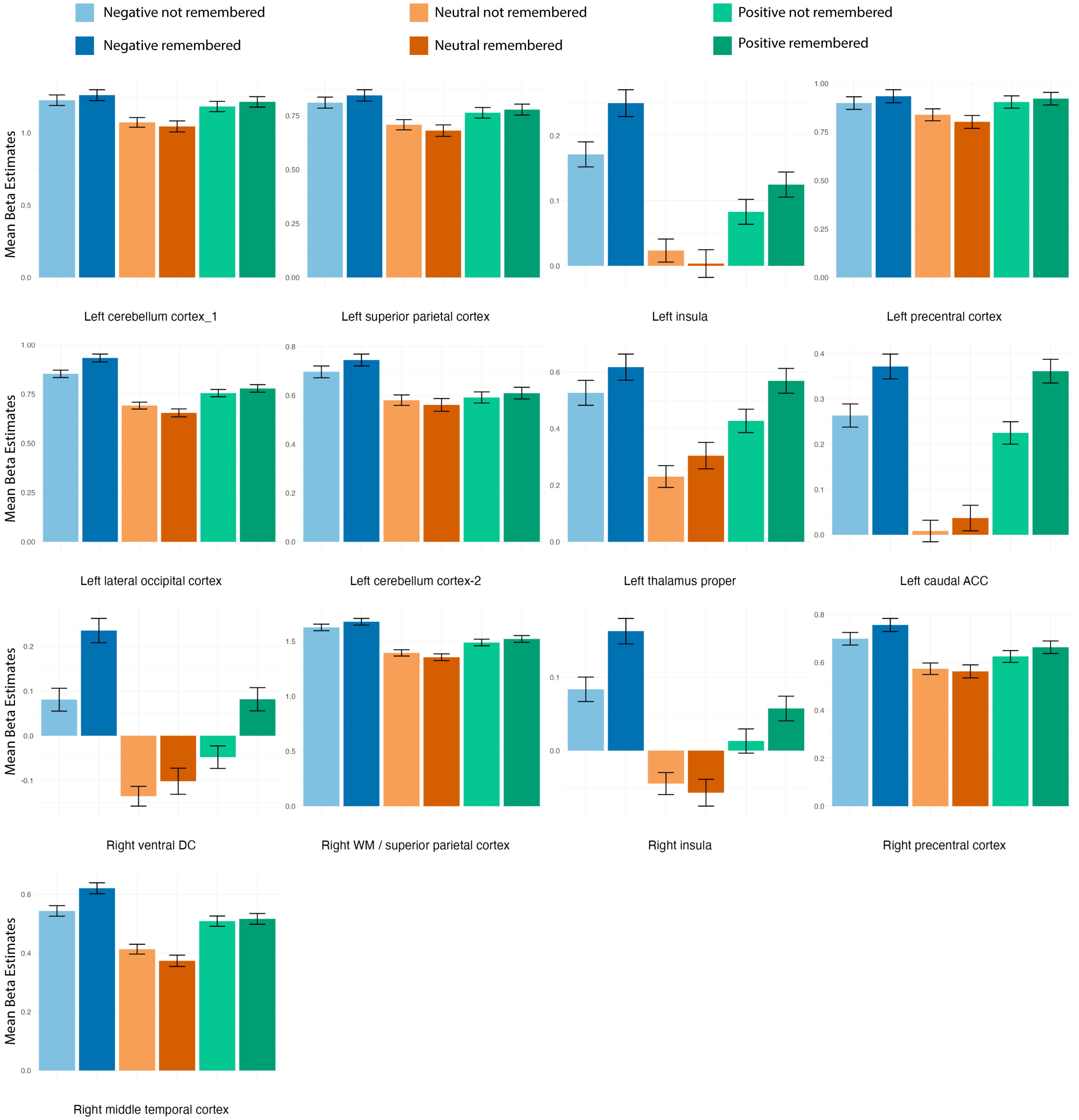

Supplementary Figure 5. Mean beta coefficient for ROIs identified in emotional memory enhancement contrast (Emotional DM > Neutral DM). Blue represents negative emotional events, orange represents neutral events, and green represents positive emotional events. Darker colours show the beta coefficient for pictures that were later remembered, and lighter colours for the ones that were not remembered. Error bars show 95% CI.

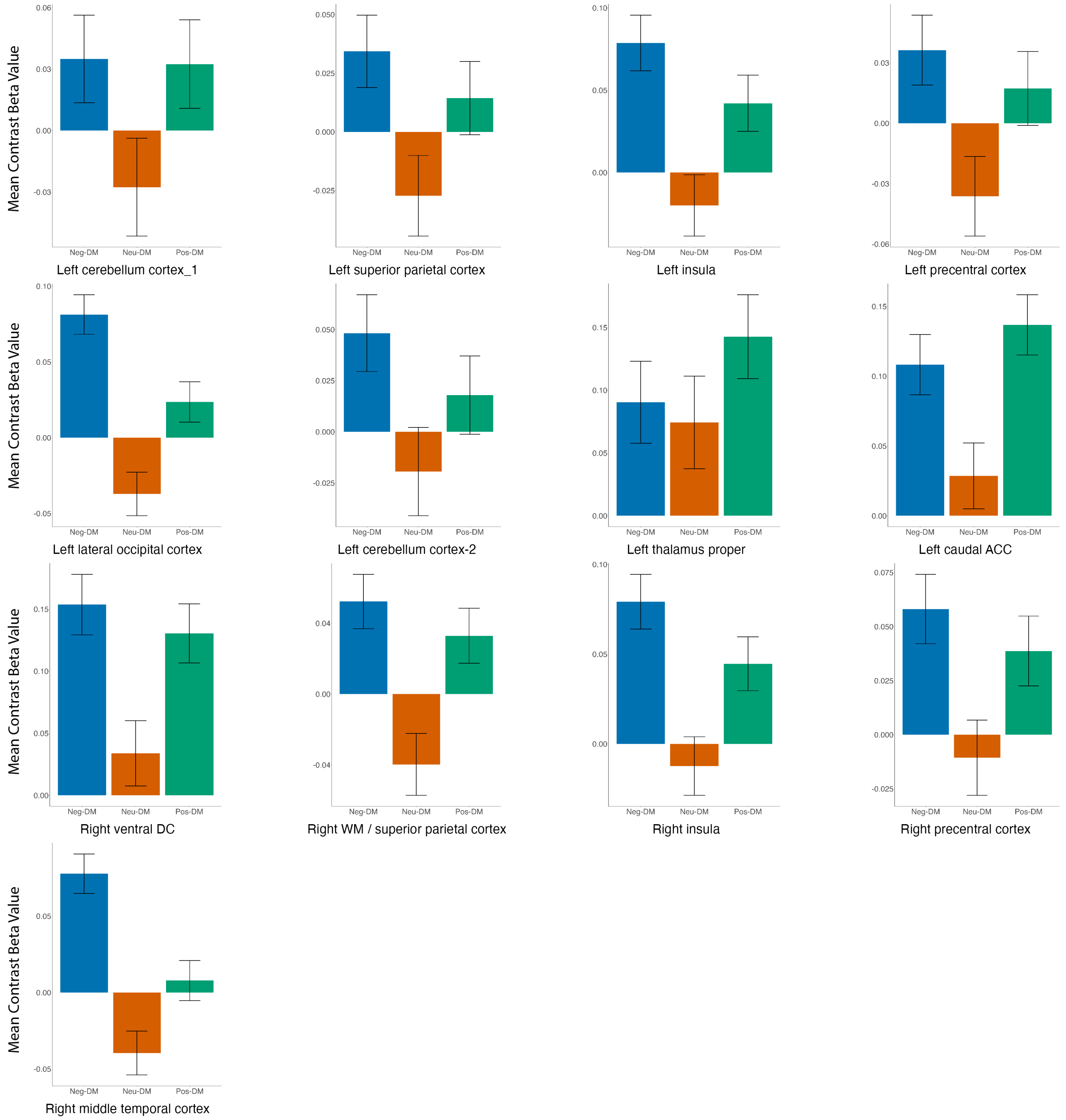

Supplementary Figure 6. Mean signal change for ROIs identified in emotional memory enhancement contrast (Emotional DM > Neutral DM). Blue represents negative DM (negative remembered > negative not remembered), orange neutral DM, and green positive DM contrast. Error bars show 95% CI. DM: difference in memory

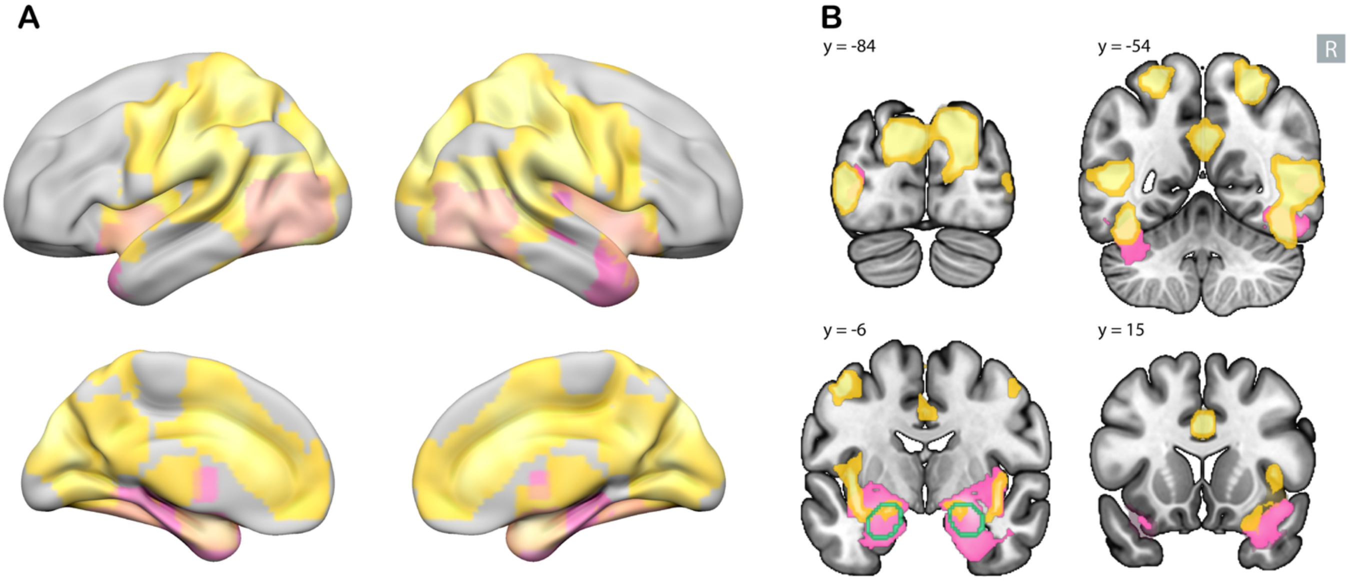

Supplementary Figure 7. Brain regions showing higher activation in Emotional DM contrast compared to neutral DM in all participants who remembered at least five pictures per valence category, irrespective of showing any emotional memory enhancement. Whole brain analysis with family-wise error (FWE)-corrected p < 0.05 and a minimum cluster size of five voxels. Subcortical regions are projected to the surface. Amygdala contour is depicted in green.

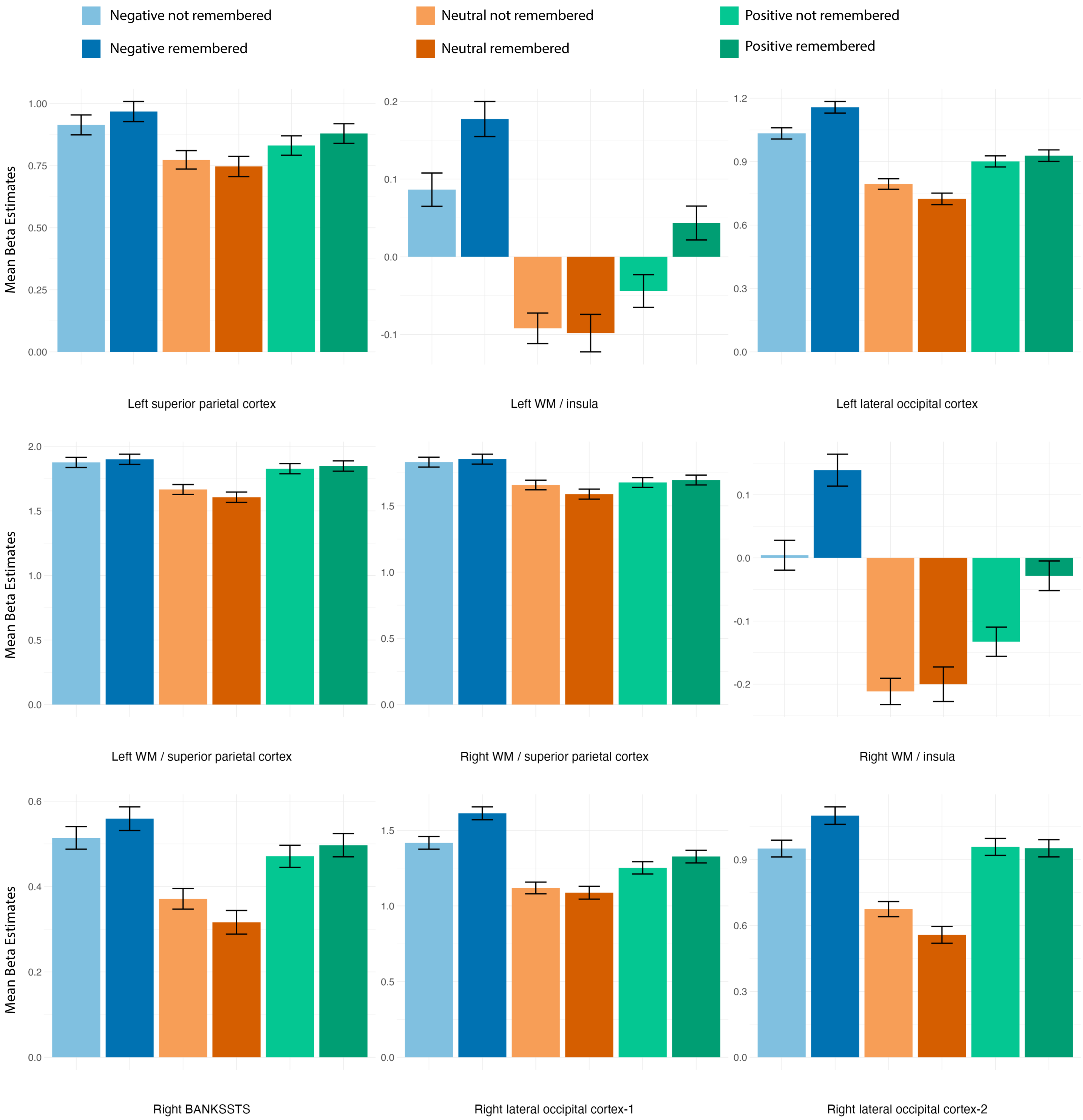

Supplementary Figure 8. Mean beta coefficient for common ROIs identified in both negative and positive emotional memory enhancement contrast ((negative DM > Neutral DM) ∩ (Pos DM > Neu DM)). Blue represents negative emotional events, orange represents neutral events, and green represents positive emotional events. Darker colours show the beta coefficient for pictures that were later remembered and lighter colours for the ones that were not remembered. Error bars show 95% CI.

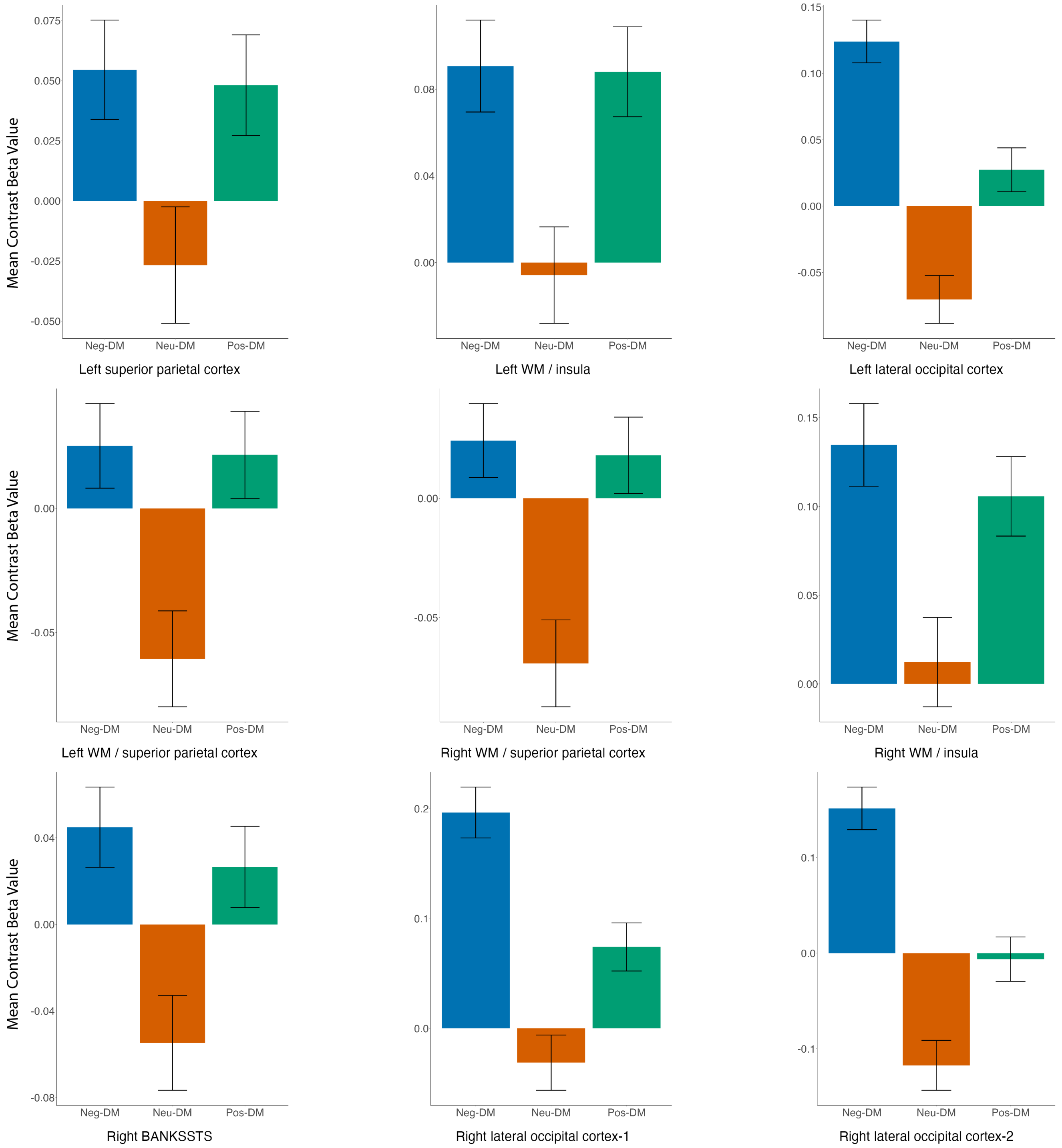

Supplementary Figure 9. Mean signal change for ROIs identified in both negative and positive emotional memory enhancement contrast ((negative DM > Neutral DM) ∩ (Pos DM > Neu DM)). Blue represents negative DM (negative remembered > negative not remembered), orange neutral DM, and green positive DM contrast. Error bars show 95% CI. DM: difference in memory. BANKSSTS: banks of the superior temporal sulcus.

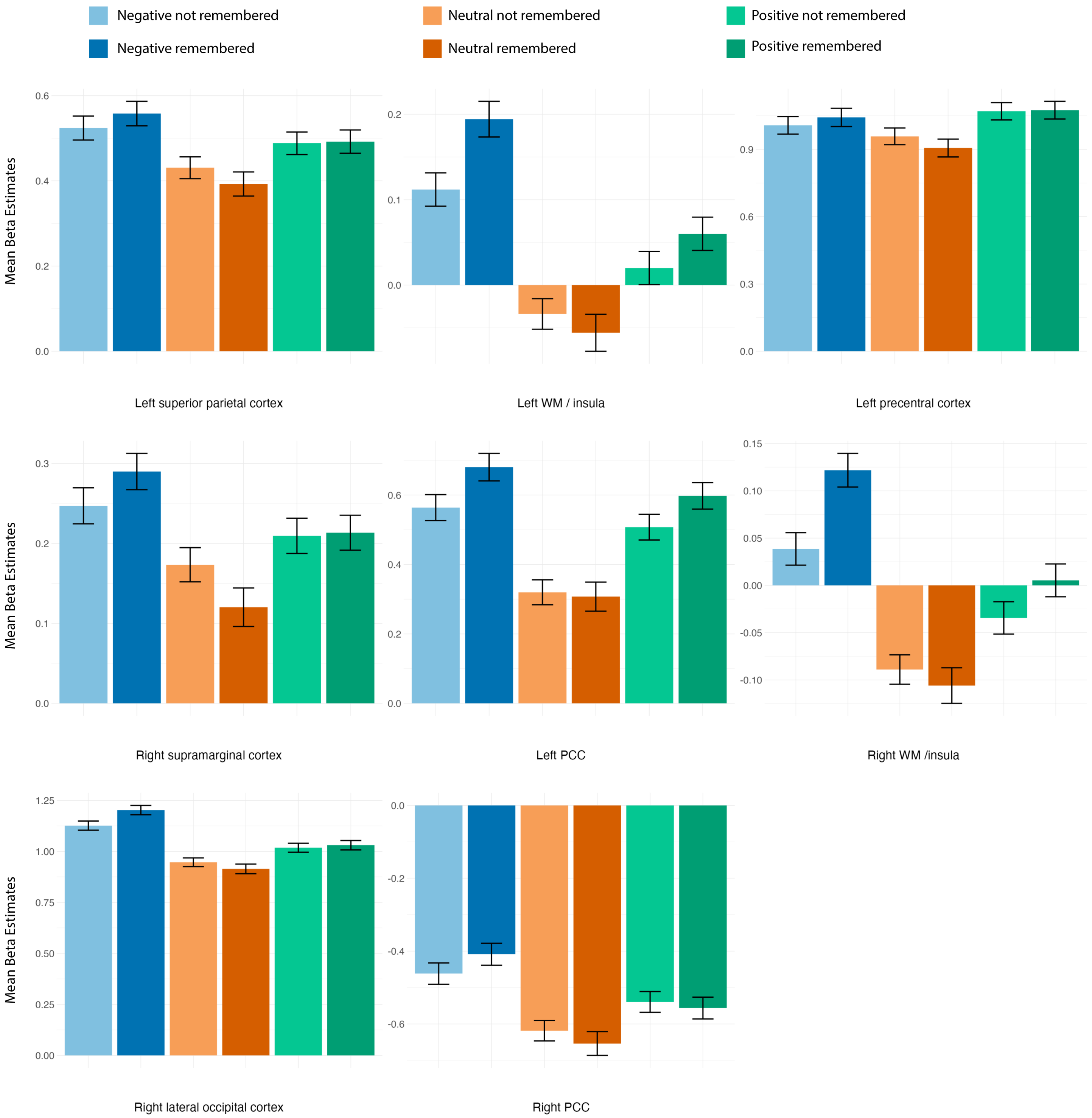

Supplementary Figure 10. The mean beta coefficient for ROIs specifically identified in negative emotional memory enhancement contrast (negative DM > Neutral DM). Blue represents negative emotional events, orange represents neutral events, and green represents positive emotional events. Darker colours show the beta coefficient for pictures that were later remembered and lighter colours for the ones that were not remembered. Error bars show 95% CI.

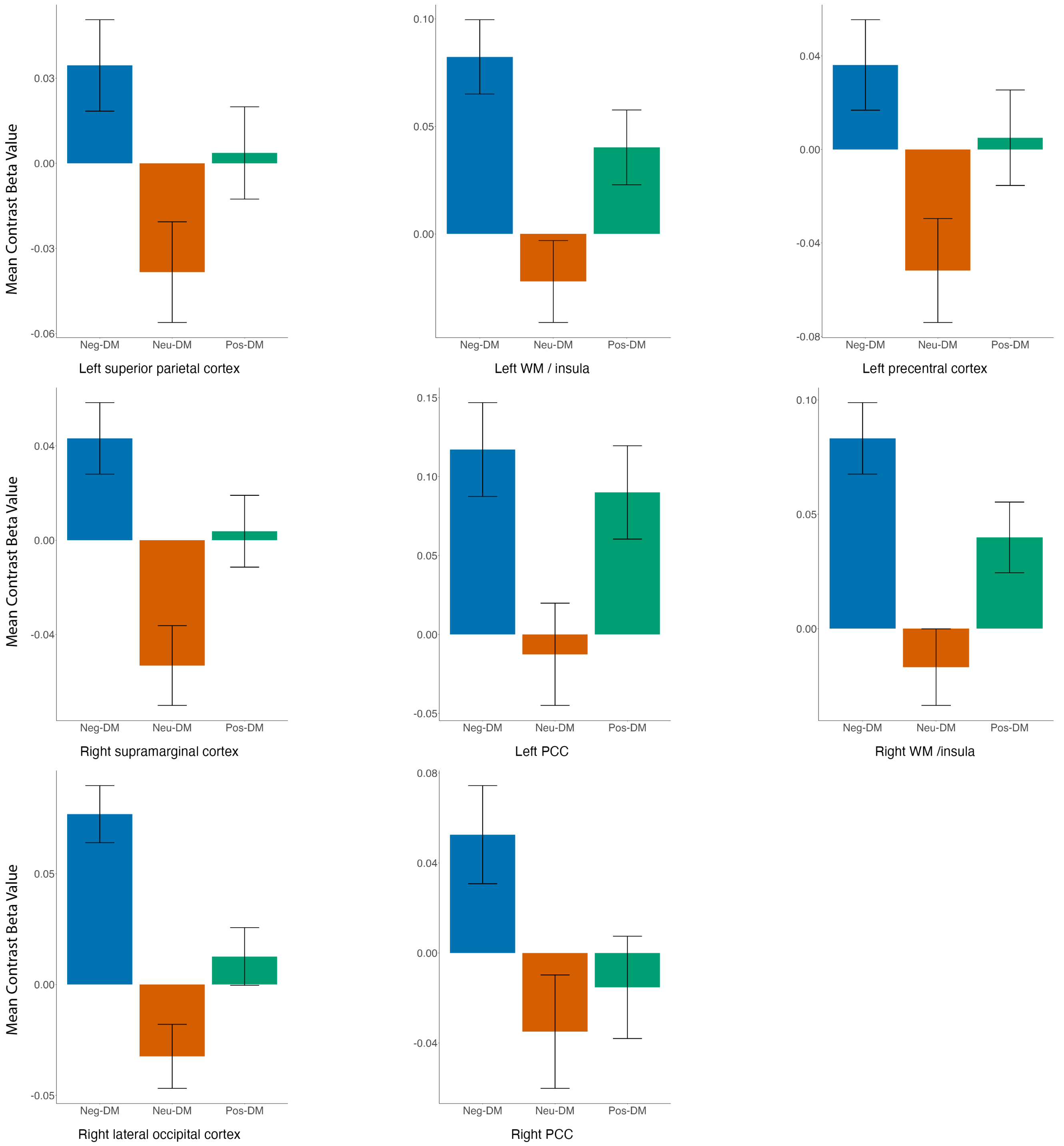

Supplementary Figure 11. Mean signal change for ROIs specifically identified in negative emotional memory enhancement contrast (negative DM > Neutral DM). Blue shows negative DM (negative remembered >negative not remembered), orange neutral DM, and green positive DM contrast. Error bars show 95% CI. DM: difference in memory

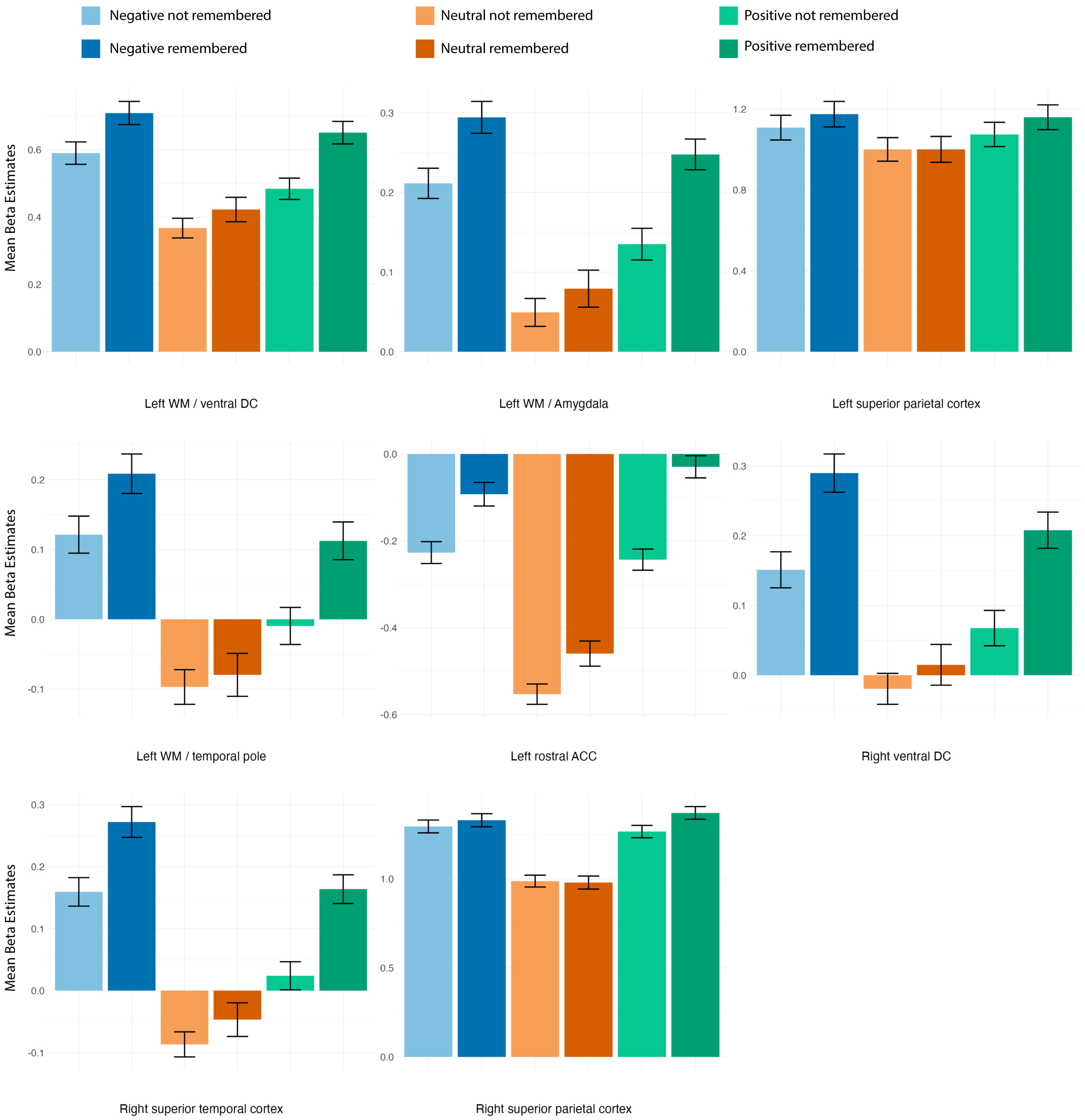

Supplementary Figure 12. Mean beta coefficient for ROIs specifically identified in positive emotional memory enhancement contrast (positive DM > Neutral DM). Blue represents negative emotional events, orange represents neutral events, and green represents positive emotional events. Darker colours show the beta coefficient for pictures that were later remembered and lighter colours for the ones that were not remembered. Error bars show 95% CI.

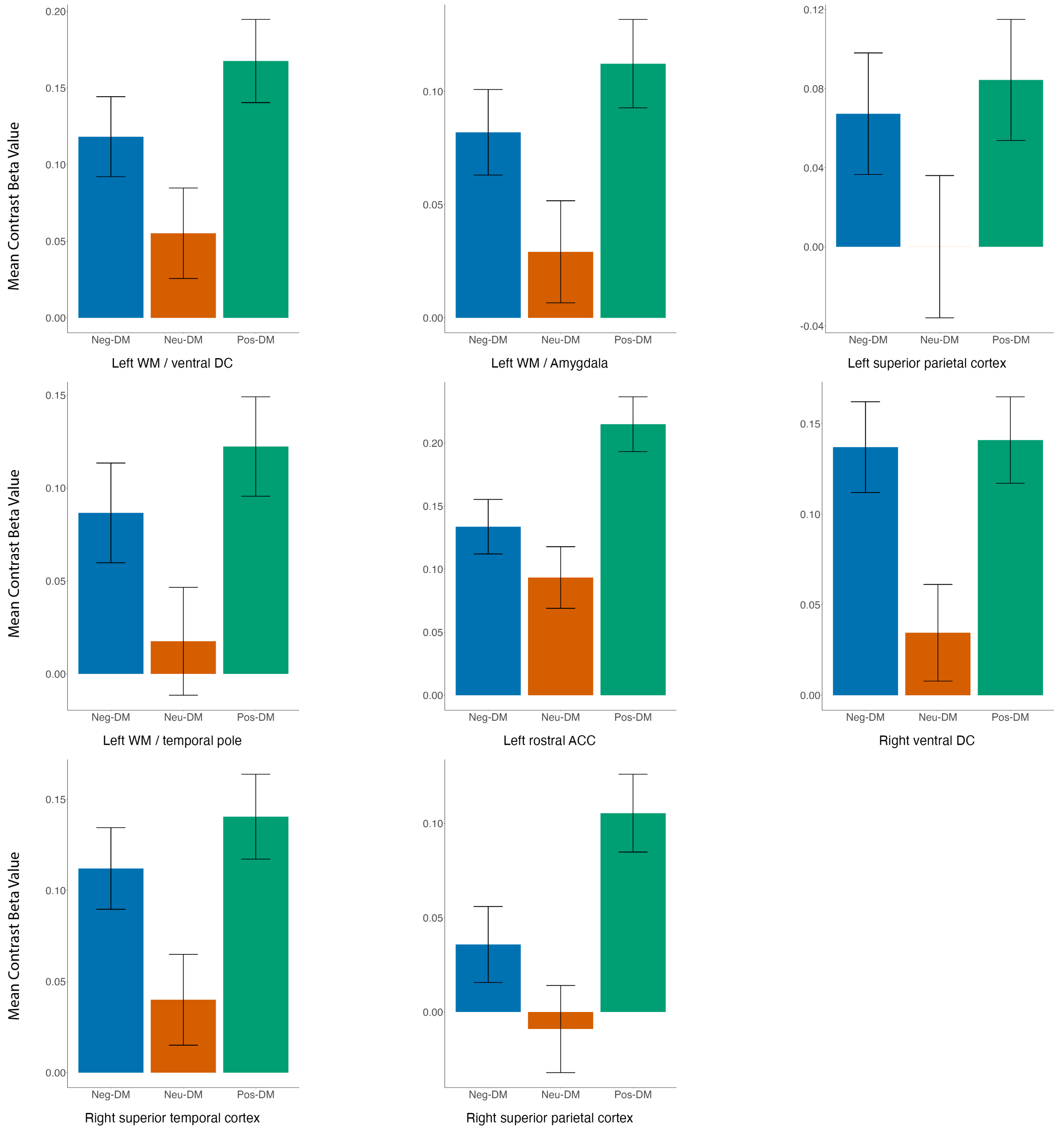

Supplementary Figure 13. Mean signal change for ROIs specifically identified in positive emotional memory enhancement contrast (positive DM > Neutral DM). Blue represents negative DM (negative remembered >negative not remembered), orange neutral DM, and green positive DM contrast. Error bars show 95% CI. DM: difference in memory

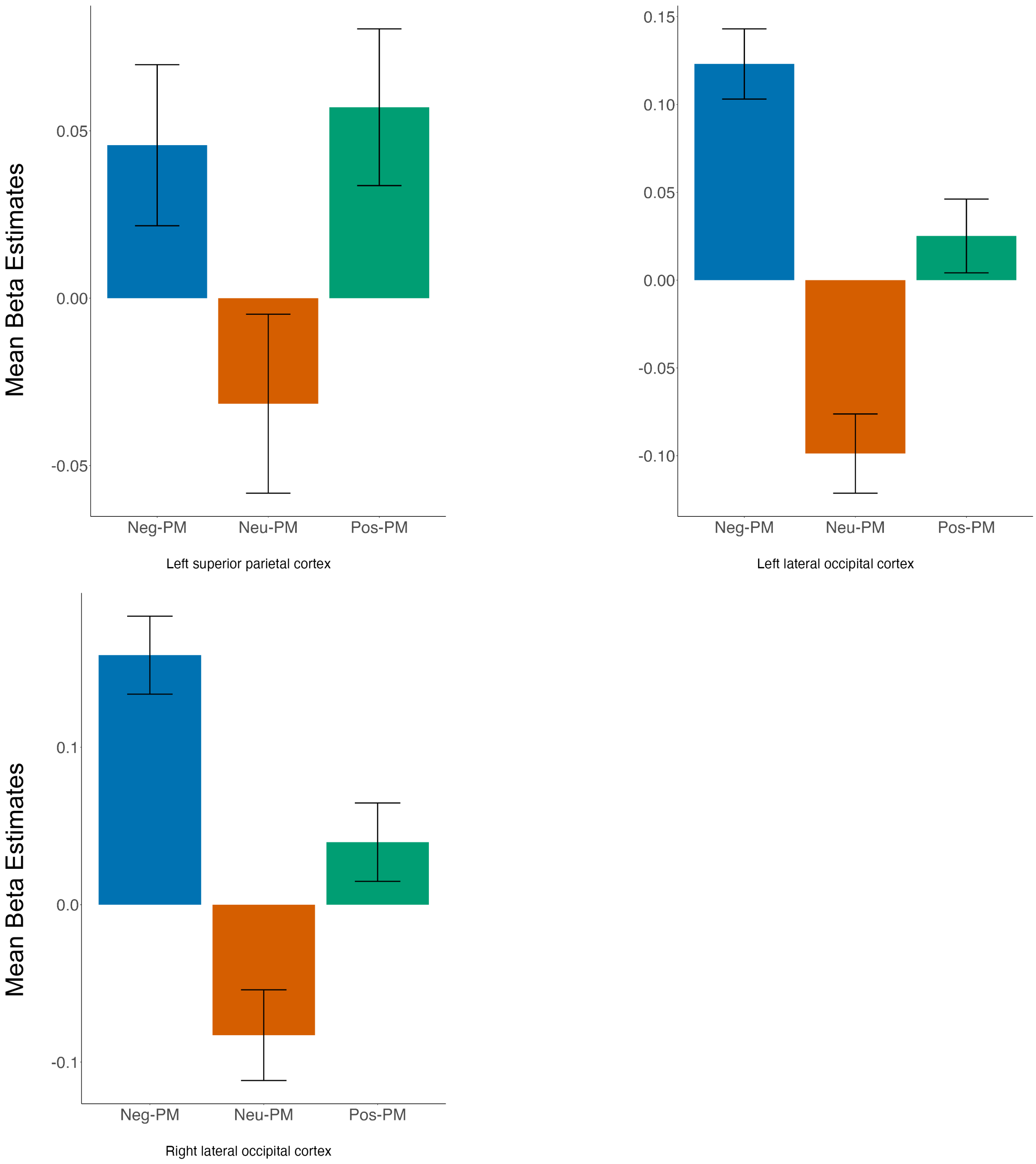

Supplementary Figure 14. The mean beta coefficient for ROIs that were identified in both negative and positive emotional memory enhancement after controlling for subjective arousal rating. Blue represents negative emotional events, orange represents neutral events, and green represents positive emotional events. Error bars show 95% CI. PM: parametric modulator

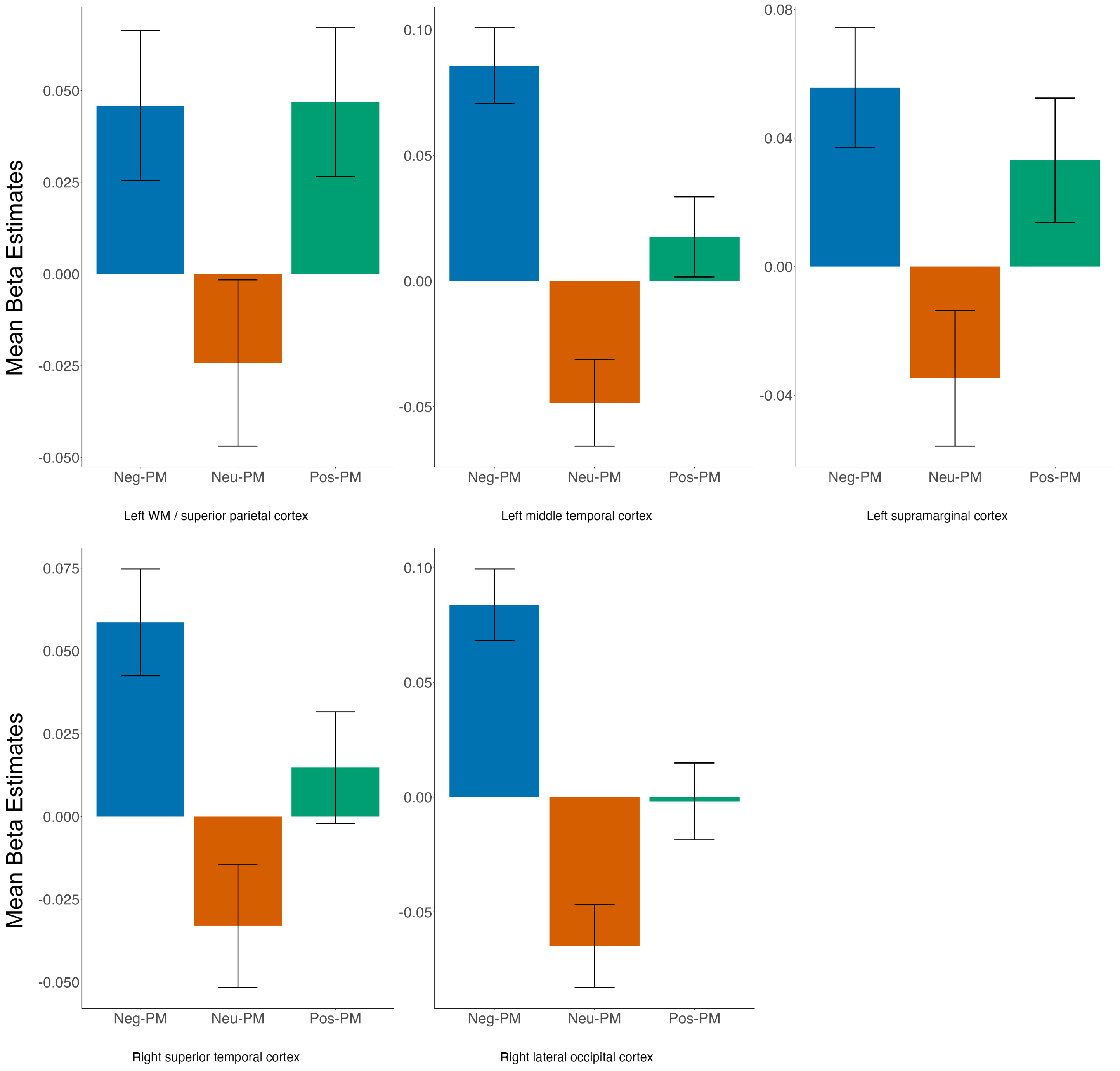

Supplementary Figure 15. The mean beta coefficient for ROIs that were identified specifically in negative emotional memory enhancement after controlling for subjective arousal rating. Blue represents negative emotional events, orange represents neutral events, and green represents positive emotional events. Error bars show 95% CI. PM: parametric modulator

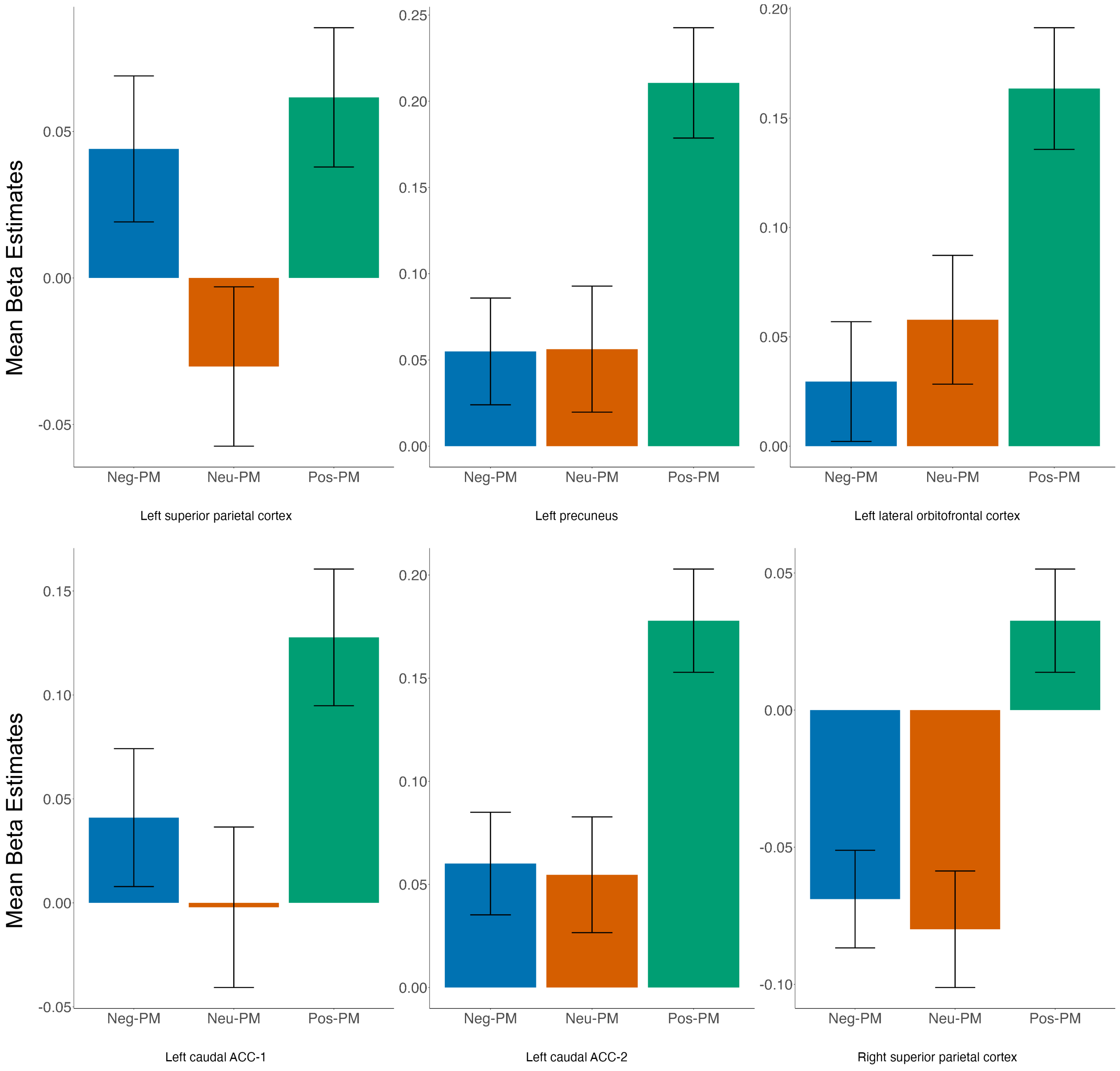

Supplementary Figure 16. The mean beta coefficient for ROIs that were identified specifically in positive emotional memory enhancement after controlling for subjective arousal rating. Blue represents negative emotional events, orange represents neutral events, and green represents positive emotional events. Error bars show 95% CI. PM: parametric modulator

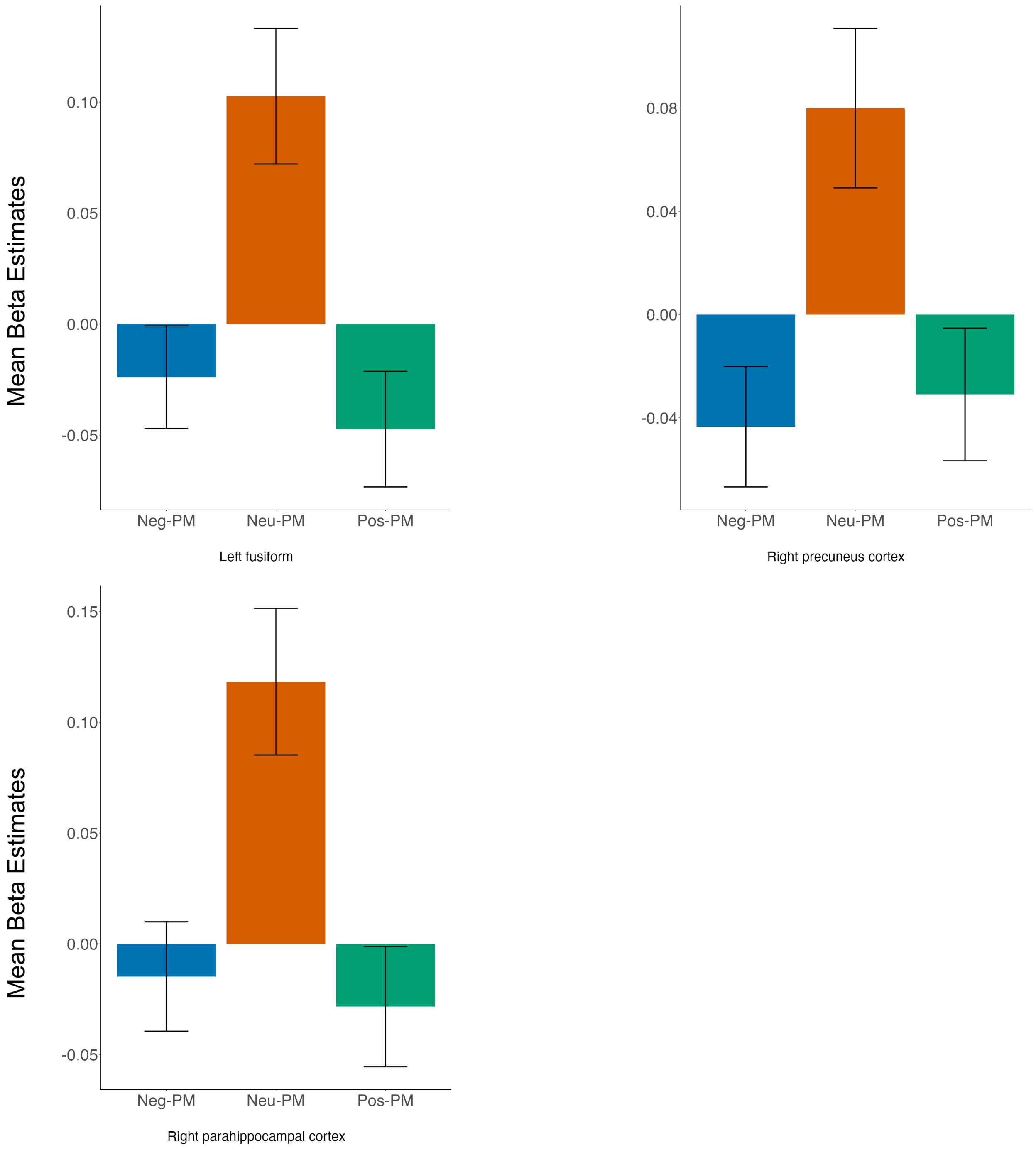

Supplementary Figure 17. The mean beta coefficient for ROIs that were identified in both (neutral memory-PM > negative memory-PM) and (neutral memory-PM > positive memory-PM) after controlling for subjective arousal rating. Blue represents negative emotional events, orange represents neutral events, and green represents positive emotional events. Error bars show 95% CI. PM: parametric modulator

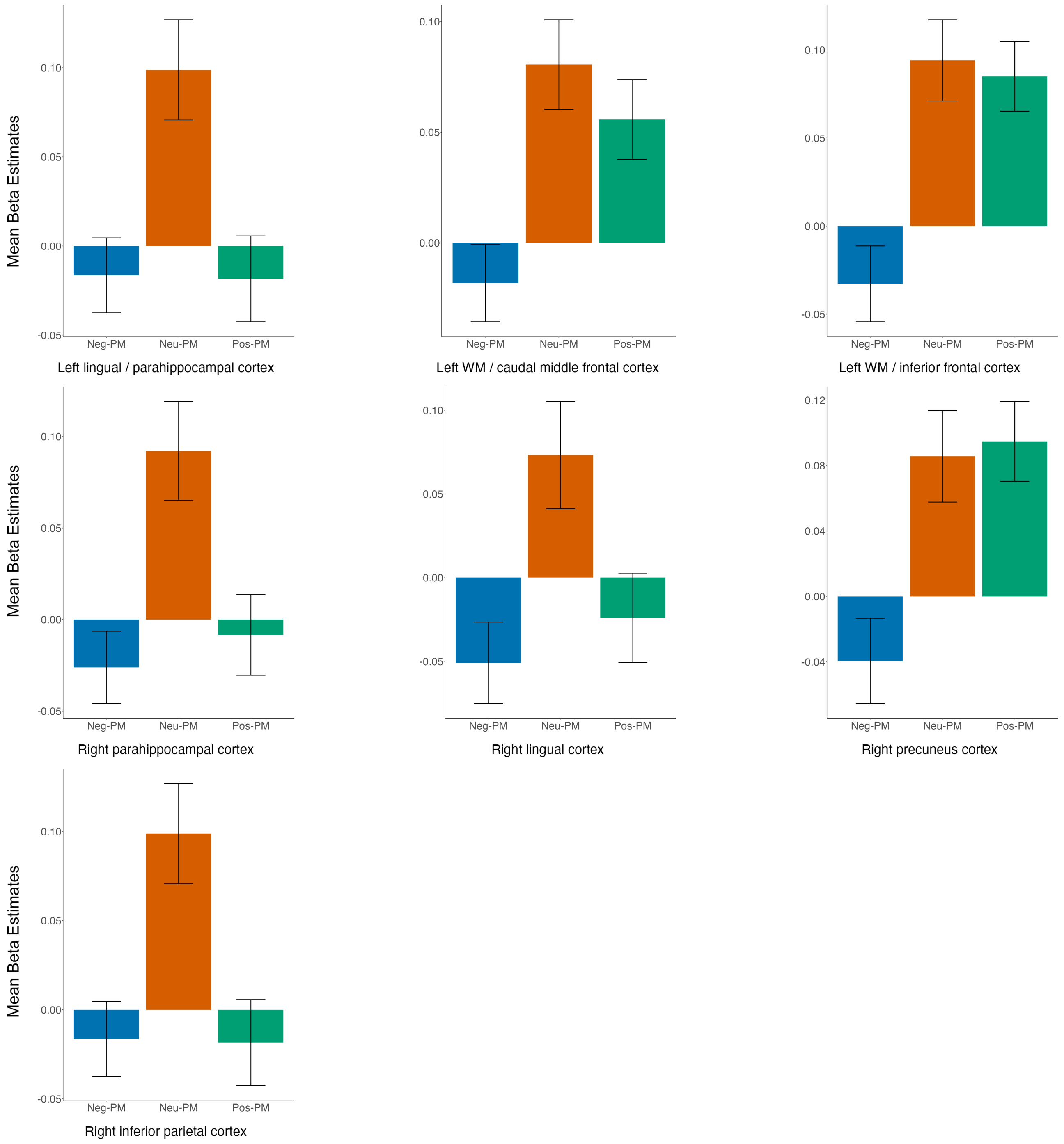

Supplementary Figure 18. The mean beta coefficient for ROIs that were identified specifically in (neutral memory-PM > negative memory-PM) after controlling for subjective arousal rating. Blue represents negative emotional events, orange represents neutral events, and green represents positive emotional events. Error bars show 95% CI. PM: parametric modulator

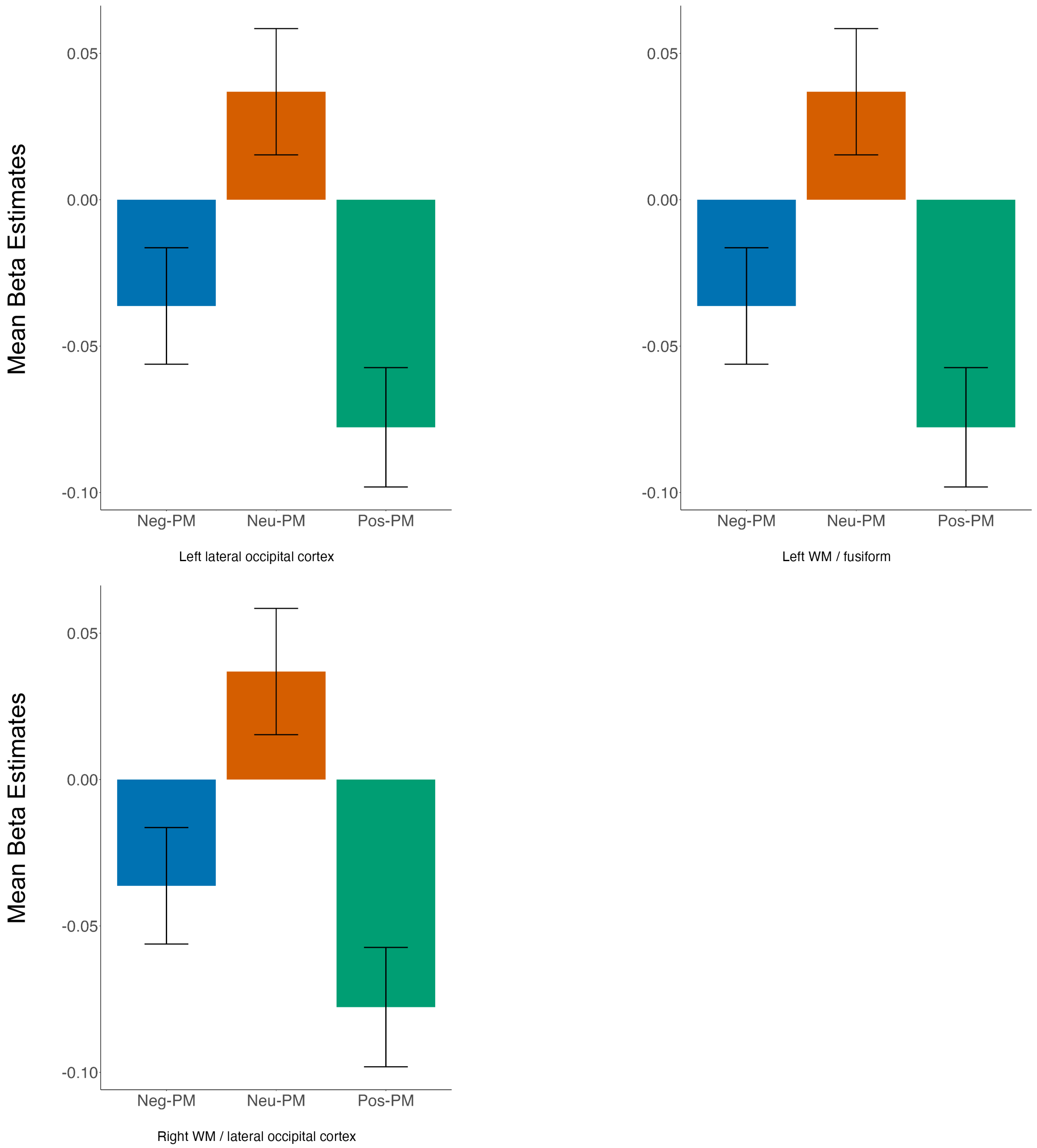

Supplementary Figure 19. The mean beta coefficient for ROIs that were identified specifically in (neutral memory-PM > positive memory-PM) after controlling for subjective arousal rating. Blue represents negative emotional events, orange represents neutral events, and green represents positive emotional events. Error bars show 95% CI. PM: parametric modulator

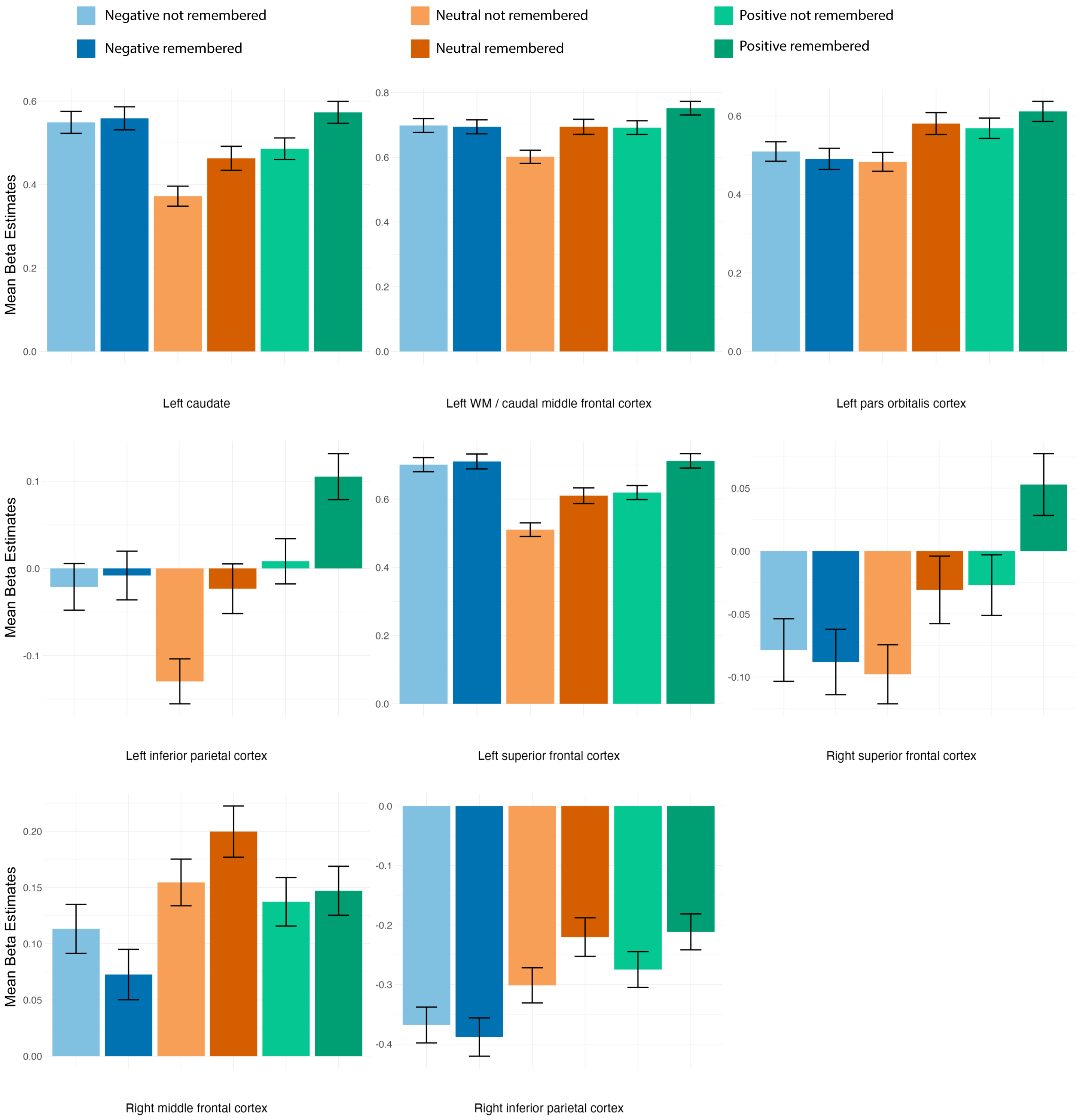

Supplementary Figure 20. Mean beta coefficient for ROIs identified specifically in neutral DM > negative DM. Blue shows negative emotional events, orange neutral events, and green positive emotional events. Darker colours show the beta coefficient for pictures that were later remembered and lighter colours for the ones that were not remembered. Error bars show 95% CI.

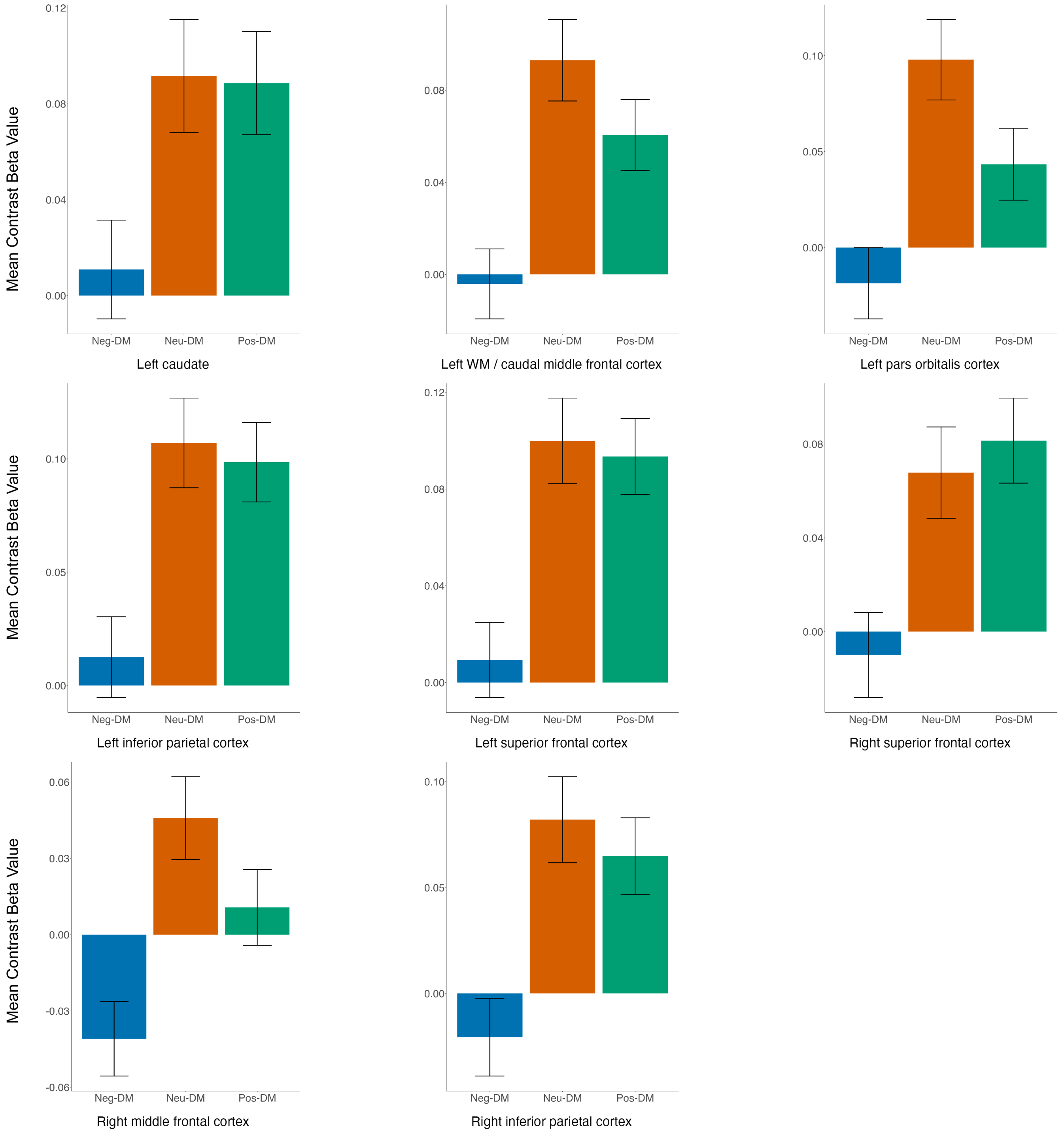

Supplementary Figure 21. Mean signal change for ROIs identified specifically in (neutral DM > negative DM). Blue represents negative DM (negative remembered >negative not remembered), orange neutral DM, and green positive DM contrast. Error bars show 95% CI. DM: difference in memory

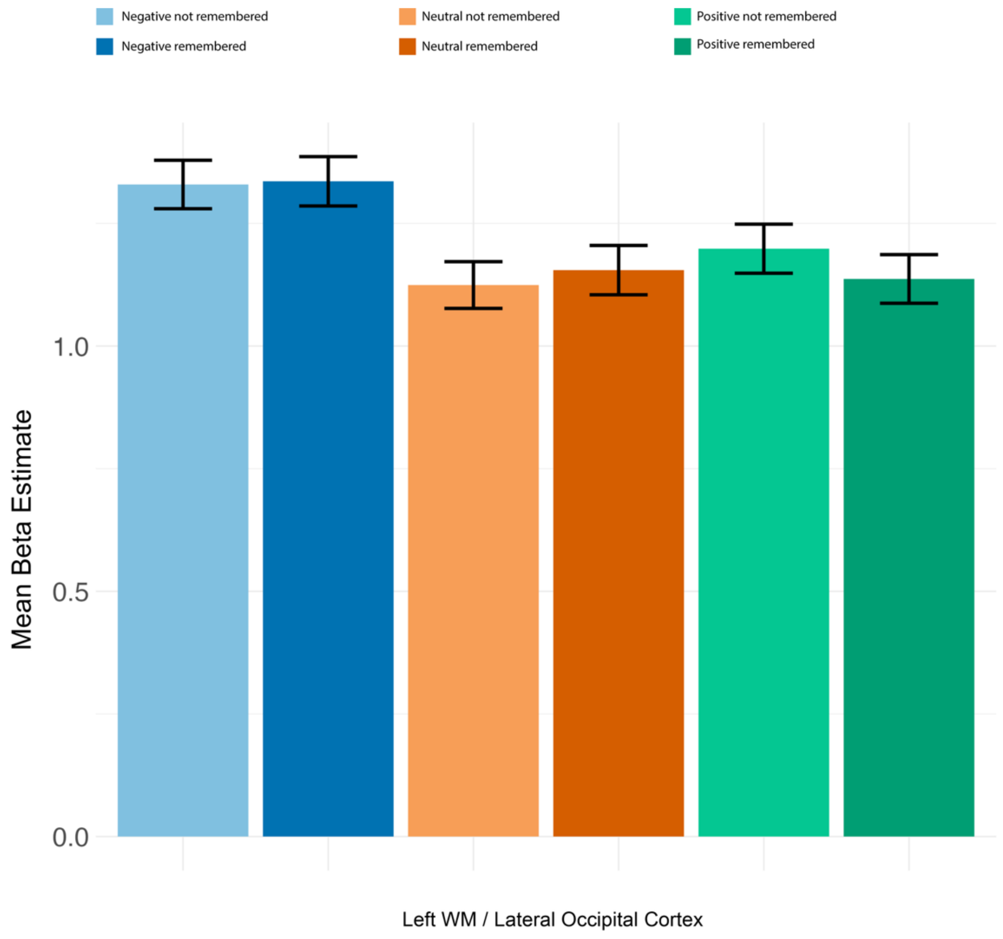

Supplementary Figure 22. Mean beta coefficient for ROIs identified specifically in neutral DM > positive DM. Blue shows negative emotional events, orange neutral events, and green positive emotional events. Darker colours show the beta coefficient for pictures that were later remembered, and lighter colours for the ones that were not remembered. Error bars show 95% CI.

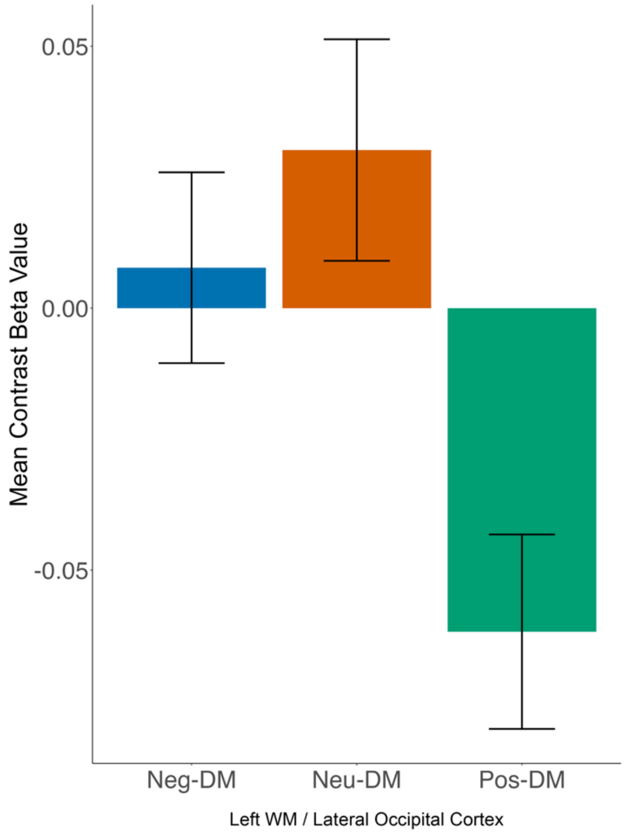

Supplementary Figure 23. Mean signal change for ROIs identified specifically in (neutral DM > positive DM). Blue represents negative DM (negative remembered >negative not remembered), orange neutral DM, and green positive DM contrast. Error bars show 95% CI. DM: difference in memory

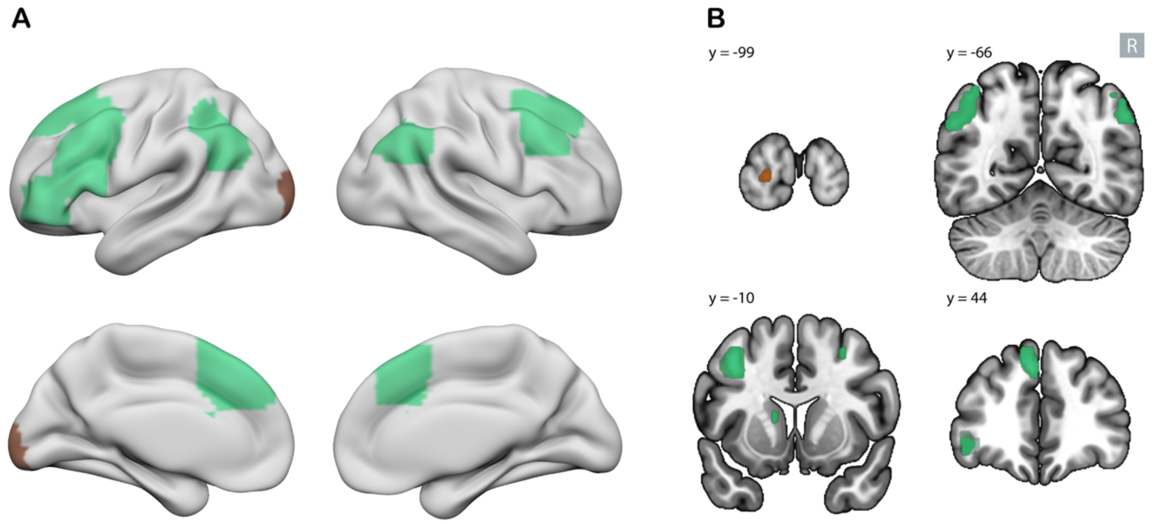

Supplementary Figure 24. Brain regions showing higher activation in neutral DM contrast compared to negative DM. *Whole brain analysis with family-wise error (FWE)-corrected $p < 0.05$ and a minimum cluster size of five voxels. Subcortical regions are projected to the surface.*

Supplementary table 1. Regions involved in emotional memory enhancement in all participants regardless of showing emotional memory enhancement

Region	ROI (glass brain)	Cluster		Peak				Network
		Size	P_{FWE}	t	x	y	z	
Left lateral occipital ctx	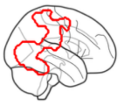	1988	<.001	14.8	-50	-74	4	VIS
Left fusiform ctx				9.4	-44	-47	-20	DATT
Left supramarginal ctx				9.4	-63	-28	28	SAL/VATT
Right middle temporal ctx	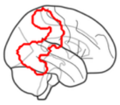	2016	<.001	13.2	50	-63	4	VIS
Right superior parietal ctx				9.3	30	-47	56	DATT
Right fusiform ctx				8.8	44	-50	-16	SAL/VATT
Right Cerebral WM / superior parietal ctx	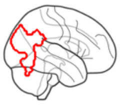	1417	<.001	8.8	16	-82	40	VIS
Left Cerebral WM				8.2	-16	-85	32	
Left Cerebral WM				7.2	-8	-85	32	
Left PCC	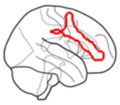	547	<.001	7.8	0	8	36	SAL/VATT
Right rostral ACC				7.0	3	38	8	Default
Left caudal ACC				6.8	0	30	16	Control
Right insula	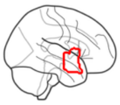	359	<.001	7.7	36	6	-12	SAL/VATT
Right insula				7.2	38	8	0	
Right ventral DC				6.4	16	-3	-12	
Left insula	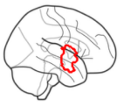	251	<.001	6.9	-38	-3	-4	SAL/VATT
Left insula				6.8	-33	0	-16	
Left amygdala				6.2	-22	-3	-16	
Left precentral ctx	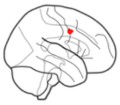	5	.006	5.4	-55	3	40	DATT
Left ventral DC	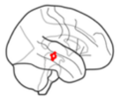	10	.002	5.4	-8	-25	-4	-
Right superior frontal ctx	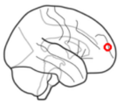	19	<.001	5.3	3	63	16	Default
Left thalamus proper	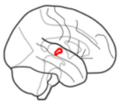	11	.002	5.3	0	-16	8	-
Right precentral ctx	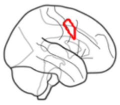	85	<.001	6.5	52	3	44	DATT
Brain stem	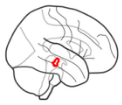	12	.001	5.1	11	-25	-12	-

Right thalamus proper	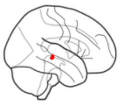	6	.005	4.8	8	-28	0	-
Left cerebellum WM	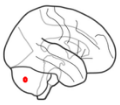	5	.006	4.7	-11	-72	-40	-

Clusters demonstrating a significantly higher association with neutral successful memory encoding than emotional successful memory encoding (family-wise error (FWE)-corrected p < 0.05 and a minimum cluster size of 5 voxels). This table presents: regions activated in neutral DM > emotional DM. Anatomical locations are based on an in-house probabilistic atlas. Secondary peak coordinates within clusters are indicated in grey. In case the voxel with peak coordinates overlaps with white matter, the closest cortical region is mentioned as well. Network abbreviations include: SAL: salient; VATT: ventral attention; WM: white matter; ctx: cortex

Supplementary table 2. Regions with higher activation in neutral successful memory encoding compared to negative and positive successful memory encoding

Region	ROI (glass brain)	Cluster Size	Cluster P_{FWE}	Peak t	Peak x	Peak y	Peak z	Network
**Section 1. Neutral DM > Negative DM**								
Left pars orbitalis ctx	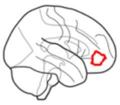	104	<.001	6.6	-44	47	-4	Control<br>Default
Left rostral middle frontal ctx				4.9	-36	58	-4	
Left superior frontal ctx	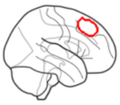	222	<.001	7.0	-6	28	44	SAL/VATT<br>Control<br>Default
Right superior frontal ctx				5.7	8	22	44	
Left cerebral WM / caudal middle frontal ctx	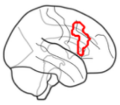	301	<.001	6.7	-38	14	48	Default
Left rostral middle frontal ctx				6.4	-47	28	28	
Left pars opercularis ctx				6.0	-52	19	12	
Right caudal middle frontal ctx	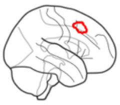	69	<.001	6.0	41	19	44	Control
Right caudal middle frontal ctx				5.8	33	16	48	
Right inferior parietal ctx	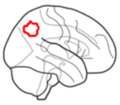	88	<.001	5.9	52	-63	40	Control
Right inferior parietal ctx				5.4	55	-55	48	
Right inferior parietal ctx				5.0	44	-63	52	
Left inferior parietal ctx	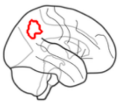	128	<.001	5.7	-52	-63	36	Default
Left cerebral WM				5.5	-41	-52	44	
Left inferior parietal ctx				5.7	-41	-60	56	
Right superior frontal ctx	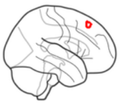	15	.001	5.4	19	33	52	Default
Left caudate ctx	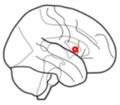	6	.005	5.0	-14	11	8	-
**Section 2. Neutral DM > Positive DM**								
Left cerebral WM / lateral occipital ctx	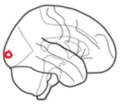	17	.004	5.2	-16	-102	0	VIS

Clusters demonstrating a significantly higher association with neutral successful memory encoding than negative successful memory encoding (family-wise error (FWE)-corrected $p < 0.05$ and a minimum cluster size of 5 voxels). This table presents: regions activated in neutral DM > negative. Anatomical locations are based on an in-house probabilistic atlas. Secondary peak coordinates within clusters are indicated in grey. In case the voxel with peak coordinates overlaps with white matter, the closest cortical region is mentioned as well. Network abbreviations include: SAL: salient; VATT: ventral attention; WM: white matter; ctx: cortex.
